# Clozapine-Induced Cardiovascular Side Effects and Autonomic Dysfunction: A Systematic Review

**DOI:** 10.3389/fnins.2018.00203

**Published:** 2018-04-04

**Authors:** Jessica W. Y. Yuen, David D. Kim, Ric M. Procyshyn, Randall F. White, William G. Honer, Alasdair M. Barr

**Affiliations:** ^1^Faculty of Medicine and Centre for Brain Health, University of British Columbia, Vancouver, BC, Canada; ^2^Department of Anesthesiology, Pharmacology and Therapeutics, University of British Columbia, Vancouver, BC, Canada; ^3^Department of Psychiatry, Faculty of Medicine Faculty of Medicine, University of British Columbia, Vancouver, BC, Canada

**Keywords:** clozapine, schizophrenia, autonomic, cardiovascular, catecholamine, heart rate, blood pressure

## Abstract

**Background:** Clozapine is the antipsychotic of choice for treatment-resistant schizophrenia and has minimal risk for extrapyramidal symptoms. Therapeutic benefits, however, are accompanied by a myriad of cardiometabolic side-effects. The specific reasons for clozapine's high propensity to cause adverse cardiometabolic events remain unknown, but it is believed that autonomic dysfunction may play a role in many of these.

**Objective:** This systematic review summarizes the literature on autonomic dysfunction and related cardiovascular side effects associated with clozapine treatment.

**Method:** A search of the EMBASE, MEDLINE, and EBM Cochrane databases was conducted using the search terms antipsychotic agents, antipsychotic drug^*^, antipsychotic^*^, schizophrenia, schizophren^*^, psychos^*^, psychotic^*^, mental ill^*^, mental disorder^*^, neuroleptic^*^, cardiovascular^*^, cardiovascular diseases, clozapine^*^, clozaril^*^, autonomic^*^, sympathetic^*^, catecholamine^*^, norepinephrine, noradrenaline, epinephrine, adrenaline.

**Results:** The search yielded 37 studies that were reviewed, of which only 16 studies have used interventions to manage cardiovascular side effects. Side effects reported in the studies include myocarditis, orthostatic hypotension and tachycardia. These were attributed to sympathetic hyperactivity, decreased vagal contribution, blockade of cholinergic and adrenergic receptors, reduced heart rate variability and elevated catecholamines with clozapine use. Autonomic neuropathy was identified by monitoring blood pressure and heart rate changes in response to stimuli and by spectral analysis of heart rate variability. Metoprolol, lorazepam, atenolol, propranolol, amlodipine, vasopressin and norepinephrine infusion were used to treat tachycardia and fluctuations in blood pressure, yet results were limited to case reports.

**Conclusion:** The results indicate there is a lack of clinical studies investigating autonomic dysfunction and a limited use of interventions to manage cardiovascular side effects associated with clozapine. As there is often no alternative treatment for refractory schizophrenia, the current review highlights the need for better designed studies, use of autonomic tests for prevention of cardiovascular disease and development of novel interventions for clozapine-induced side effects.

## Introduction

Clozapine (CLZ) is the antipsychotic drug of choice for treatment-resistant schizophrenia, displaying superior efficacy in an estimated 30% of patients who are persistently unresponsive to other antipsychotic drugs (Kane et al., 1988; Meltzer, 1997; Honer et al., 2015). CLZ has been shown to effectively reduce suicidality in patients with treatment-resistant schizophrenia (Meltzer and Okayli, 1995; Reinstein et al., 2002) and is the sole agent approved by the U.S. Food and Drug administration to manage suicidal behavior in persons with schizophrenia or schizoaffective disorder (Citrome et al., 2016). Compared to most first generation antipsychotics (FGAs) and many second generation antipsychotics (SGAs), CLZ has minimal risk for extrapyramidal symptoms (EPS) (Lindström, 1988; Casey, 1989) and does not induce hyperprolactinemia (Kane et al., 1981; Melkersson, 2005). In a study comparing treatment duration between FGAs and SGAs as an indicator of overall effectiveness, CLZ was an important factor in lengthening the time to medication discontinuation of SGAs as a group (Ascher-Svanum et al., 2006). Furthermore, fewer relapses and rehospitalization rates are reduced with CLZ use (Essock et al., 1996; Conley et al., 1999; Essali et al., 2009). The abovementioned superiority has been attributed to CLZ's diverse receptor binding profile (Bymaster et al., 1996; Nasrallah, 2008), although the exact mechanism remains unknown. CLZ has a high-affinity for adrenergic receptors α_1_, α_2_ (Kalkman et al., 1998), dopamine receptors D_1_ (Kalkman et al., 1998), D_2_ (Masri et al., 2008), and D_4_ (Van Tol et al., 1991), serotonin receptors 5-HT_6_ (Monsma et al., 1993) and 5-HT_7_ (Shen et al., 1993), muscarinic receptors (Richelson and Souder, 2000) and histaminergic receptors H_1_ and H_3_ (Bymaster et al., 1997). CLZ may also show superior efficacy in treating psychostimulant drug-induced psychosis (Seddigh et al., 2014), which is associated with neuropsychiatric and structural brain changes similar to those of schizophrenia (Tang et al., 2015; Willi et al., 2016a,b).

Any therapeutic benefit from CLZ treatment, however, must take into consideration the high incidence of non-EPS side-effects associated with the drug (Andreazza et al., 2015; Thornton et al., 2015; Tse et al., 2015; Lee et al., 2016). An estimated 10–20% of eligible patients are prescribed with CLZ in the U.S., a number indicative that CLZ is vastly underutilized due to concern for its side effects (Meltzer, 2012; Kar et al., 2016). One of the most concerning side effects of CLZ is agranulocytosis, which occurs in approximately 1% of patients receiving CLZ (Alvir et al., 1993) and only rarely with other antipsychotics (Vila-Rodriguez et al., 2013). Critically, there is a large body of evidence of CLZ's high propensity to induce cardiometabolic side effects, such as orthostatic hypotension (Mackin, 2008), tachycardia (Safferman et al., 1991; Young et al., 1998), myocarditis (Kilian et al., 1999; Merrill et al., 2005), dyslipidemia (Olfson et al., 2006; Procyshyn et al., 2007, 2009), weight gain and obesity (Henderson et al., 2000; Whitney et al., 2015). Increased cardiovascular risk is of concern, especially when coronary heart disease (CHD) is the leading cause of premature death in schizophrenia (Hennekens et al., 2005). Indeed, a 10 year naturalistic study of CLZ-treated patients estimated the 10 year mortality from cardiovascular disease (CVD) to be at 9% (Henderson et al., 2005). While the increased liability for cardiovascular risk in patients with schizophrenia has been linked to lifestyle habits such as smoking, physical inactivity/sedentary behavior and unhealthy diet (McEvoy et al., 2005; Bobes et al., 2010; Lang et al., 2013; Fredrikson et al., 2014), antipsychotic use, in particular CLZ, has increased the risk of sudden cardiac deaths (Modai et al., 2000; Ray et al., 2009). The exact cause is unknown, however, myocarditis (La Grenade et al., 2001; Fineschi et al., 2004) and pulmonary embolism (Walker et al., 1997) resulting from CLZ treatment have been implicated.

The abovementioned cardiovascular side effects stem largely from autonomic dysregulation, namely antagonism of adrenergic and cholinergic receptors that influence autonomic function (Leung et al., 2012). Briefly, cardiovascular function is regulated by the autonomic nervous system (ANS), which is further divided into the sympathetic nervous system (SNS) and the parasympathetic nervous system (PNS). The SNS is comprised of neurons that relay impulses to and from the central nervous system (CNS). The SNS efferent neurons consist of 2 groups of neurons, the preganglionic and postganglionic sympathetic neurons. Preganglionic sympathetic neurons originate from the CNS and synapse with postganglionic sympathetic neurons at peripheral sympathetic ganglia either located in the sympathetic chain adjacent to the spinal cord or near target organs (Jänig, 2006; Triposkiadis et al., 2009). The postganglionic sympathetic neurons release the neurotransmitter norepinephrine (NE) upon stimulation, which in turn stimulates adrenergic receptors to influence physiological functions such as increasing heart rate (HR) and blood pressure (BP) (Jänig, 2006; Triposkiadis et al., 2009). The PNS acts in opposition to the SNS to achieve cardiac homeostasis, although there are exceptions to this conventional view. As reviewed by Paton et al., parasympathetic and sympathetic nerves innervating the heart can act in synergy during reflex responses including the startle, diving and somatic nociceptor reflexes (2005). The combined contribution from both the PNS and SNS to the heart can result in arrhythmia and possibly contribute to cardiac pathophysiology, a subject that warrants further electrophysiological experiments (Paton et al., 2005).

Schizophrenia has been associated with autonomic dysregulation, possibly due to imbalance of sympathetic and vagal control (Bär et al., 2007, 2010; Chang et al., 2009). Antipsychotic medication can also influence autonomic function (Buckley and Sanders, 2000), with CLZ consistently viewed as one of the SGAs with the highest risk for CVD and Type 2 diabetes (Henderson et al., 2005; De Hert et al., 2011). Risk for CVD was investigated in a study conducted by Henderson et al. (2005), where medical records of 96 patients with schizophrenia were screened over 10 years for metabolic and cardiovascular anomalies as a result of CLZ treatment. Results indicate time-dependent significant increases in body mass index (BMI) and average weight gain of 30 lbs, as well as an increase in the number of cardiovascular risk factors that were assessed yearly, including hypertension, smoking and cholesterol or triglyceride levels. Multiple cardiovascular-related adverse events were also reported in this study (*n* = 11), with 7 deaths, 3 myocardial infarctions and 1 cerebrovascular accident (Henderson et al., 2005). An especially grave adverse effect of CLZ is myocarditis. Haas et al. (2007) identified 116 case reports for myocarditis-related fatality in CLZ-treated patients. Of these cases, 17 failed to recover and 12 patients died from suspected myocarditis (Haas et al., 2007). The authors noted myocarditis occurs within 4 weeks of administering CLZ at 100–450 mg/day, with a prevalence of 0.7–1.2% in CLZ-treated patients.

Given the evidence of cardiovascular complications resulting from CLZ treatment, it is imperative to understand how CLZ influences cardiovascular function and identify methods to balance the costs and benefits of prescribing CLZ. This systematic review is a comprehensive search of the literature for cardiovascular incidents resulting from CLZ use, with a specific emphasis on the ANS. We aim to compile reports of CLZ-induced cardiovascular side effects from past research, identify methods for early detection and describe clinical interventions to minimize CVD risk in CLZ-treated patients.

## Methods

The EMBASE, MEDLINE and EBM Cochrane databases were searched using the following key words: antipsychotic agents, antipsychotic drug, antipsychotic, schizophrenia, psychotic, mental illness, mental disorder, neuroleptic, cardiovascular, cardiovascular diseases, clozapine, clozaril, autonomic, sympathetic, catecholamine, norepinephrine, noradrenaline, epinephrine and adrenaline. The date range was set to 1959 (first introduction of CLZ) to March 2, 2016. Case reports, animal studies and clinical studies published in English were included. Additional records were identified from reference lists and independent searches. Human studies were excluded if subjects were under 18 years of age. The primary author conducted all screening and extraction of relevant articles from the search. Due to the mixed nature and amount of articles identified, the authors conducted a systematic review instead of a meta-analysis for better representation of the available literature.

## Results

**Figure 1:**
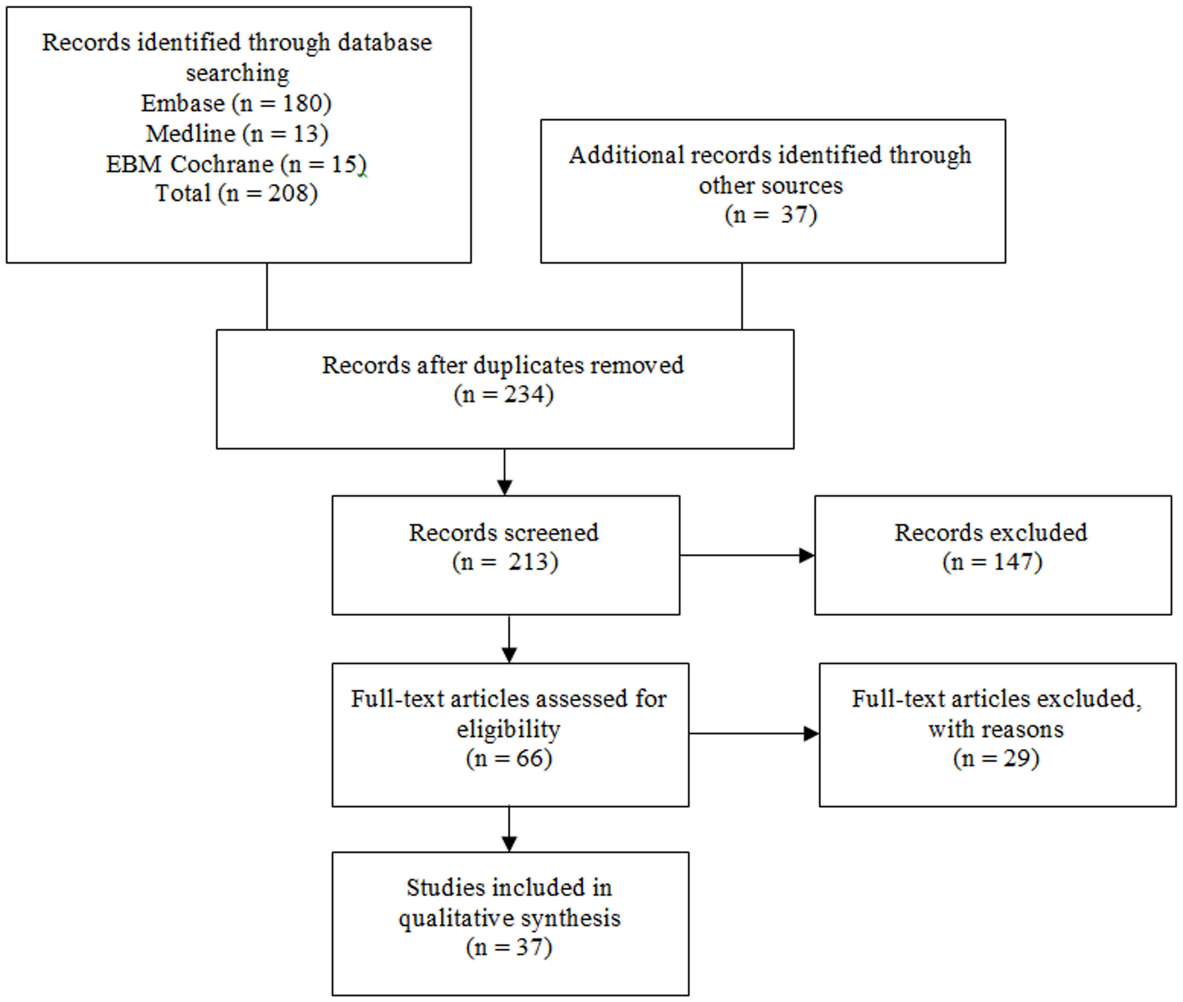
Flow diagram for identification of studies. Original studies discussing clozapine, cardiovascular disease and autonomic function are reviewed. Keywords used: antipsychotic agents, antipsychotic drug^*^, antipsychotic^*^, schizophrenia, schizophren^*^, psychos^*^, psychotic^*^, mental ill^*^, mental disorder^*^, neuroleptic^*^, cardiovascular^*^, cardiovascular diseases, clozapine^*^, clozaril^*^, autonomic^*^, sympathetic^*^, catecholamine^*^, norepinephrine, noradrenaline, epinephrine, and adrenaline.

The literature search identified 208 studies as outlined in Figure 1 (*n* = 180 from Embase, *n* = 13 from MEDLINE and *n* = 15 from EBM Cochrane). Additional records were identified from other sources (gray literature and reference lists) (*n* = 33) and 11 duplicates were found. Twenty-one studies were excluded due to publication in a language other than English and 147 studies were excluded for irrelevance based on screening of abstracts and titles. The remaining 66 full-text articles were assessed and 29 full-text articles were excluded with reasons (i.e. CLZ was not administered, the study did not involve patients with schizophrenia, autonomic function was not discussed, human subjects were under 18 years of age and/or the article is a review). The final 37 articles comprised of case reports and studies evaluating the effects of CLZ on autonomic function. See Table 1 in Supplementary Material for full summary of results.

### Autonomic function tests

Various tests have been developed to assess autonomic function and screen for autonomic neuropathies (see Table 2 in Supplementary Material for summary). These tests evaluate HR and/or BP response to stimuli, thus reflecting sympathetic and parasympathetic regulation of cardiovascular function. The following sections summarize commonly used tests for non-invasive assessment of autonomic function.

### Heart rate variability

Spectral analysis of heart rate variability (HRV) is a noninvasive, beat-to-beat analysis of fluctuations in HR (Wheeler and Watkins, 1973). HRV assesses both sympathetic and parasympathetic function via time and frequency domain measurements (1996). Time domain measures are based on interbeat HR or HR between successive cycles (Stein et al., 1994). One simple measure based on interbeat HR is the standard deviation of all NN intervals (SDNN), where NN refers to normal-to-normal intervals or intervals between successive QRS complexes (1996). SDNN is commonly calculated over a 24 h period and reflects the total HRV for the duration of the recording (1996). Each measure of interbeat variability reflects individual contributions from the parasympathetic, sympathetic and renin-angiotensin systems to sinus rhythm control (outlined in Table 2 in Supplementary Material, Akselrod et al., 1981). The second class of time domain measurements is based on interval differences between successive cycles and a typical example is the square root of the mean squared differences of successive NN intervals (RMSSD). This measure is an estimate of the short-term components of HRV and generally reflects vagal activity (Stein et al., 1994). Frequency domain measures describe how variance is distributed as a function of frequency, generally divided into short-term and long-term spectral components (1996). Short-term spectral recordings consist of high frequency (HF), low frequency (LF), and very low frequency (VLF) components, where HF is generally linked to parasympathetic regulation whereas LF is mediated by both the SNS and PNS (Stein et al., 1994). Long term recordings measure the variance of all NN intervals in a 24 h period (1996). Reduced HRV has been linked to increased risk of mortality from cardiac disease and predicts risk for future cardiac events, thus generating an increasing interest in the clinical value of HRV (Tsuji et al., 1996; La Rovere et al., 1998; Nolan et al., 1998; Dekker et al., 2000).

Cohen et al. (2001b) compared HRV in patients with schizophrenia treated with CLZ (*n* = 21) to those treated with haloperidol (HAL, *n* = 18) or olanzapine (OLA, *n* = 17) and found CLZ induced tachycardia while significantly lowering the total variability of HR. They also found an increased LF/HF ratio in CLZ-treated patients, indicative of increased sympathetic activity associated with CLZ (Cohen et al., 2001a). Spectral analysis can also be applied toward respiratory sinus arrhythmia (RSA), derived from the HF band of HRV, and is a measure of parasympathetic activity (Mathewson et al., 2012). The relationship between RSA and cognitive function was assessed in CLZ-treated patients. Results show CLZ worsened performance in the Wisconsin Card Sorting Test (WCST) and significantly decreased parasympathetic control, compared to healthy controls and patients treated with other antipsychotics. However, the inverse relationship between parasympathetic control and number of perseverative errors committed in patients with schizophrenia did not change with CLZ use (Mathewson et al., 2012).

CLZ's anti-cholinergic effects were investigated in a study comparing HRV in patients with schizophrenia receiving antipsychotics with high affinity for muscarinic receptors (HMA) to those with low affinity (LMA), with CLZ belonging to the HMA group (Huang et al., 2013). Of the 55 subjects recruited, 28 received HMA and displayed perturbed sympathetic and parasympathetic regulation. Correlation and multiple linear regression analyses revealed muscarinic receptor affinity was negatively associated with HRV profiles, namely HF, LF, and TP (Huang et al., 2013). Due to suppression of both the LF and HF components, the LF/HF ratio in the HMA group was not significantly different from that of the LMA group. Interestingly, the authors show the suppression of HRV in the HMA group was still present after controlling for metabolic factors, suggesting the metabolic syndrome does not significantly influence cardiovascular function. The study repeated with a larger sample size, in particular with CLZ-treated patients, will help clarify the role of antipsychotics with high metabolic liability in cardiovascular function. CLZ, as a HMA, may also contribute to increased motivation for smoking (Barr et al., 2008), which adds to cardiovascular burden.

Tümüklü et al. (2008) studied CLZ-induced arrhythmia in patients with schizophrenia over a course of 10 weeks. Parameters measured included HRV, QT dispersion (myocardial repolarization) and late potentials (delayed ventricular conduction). Both QT dispersion and late potentials displayed an increasing trend following CLZ treatment, yet the changes were statistically insignificant. HRV, however, was suppressed in CLZ-treated patients, with LF/HF significantly influenced by age and gender (i.e., patients <35 years old, female patients). Decreased sympathetic activity was attributed to CLZ's potent antagonism of muscarinic and α-adrenergic receptors (Tümüklü et al., 2008).

Reduced HRV from CLZ treatment was also noted in a prospective clinical study comparing the effects of amisulpride, OLA, CLZ, and sertindole on HRV (Agelink et al., 2001). CLZ was the only neuroleptic to significantly reduce parasympathetic activity, with half of the CLZ-treated patients showing signs of suppressed HRV (Agelink et al., 2001). Interestingly OLA, a SGA with comparable anticholinergic properties with CLZ *in vitro*, did not demonstrate the same extent of drug-induced tachycardia and parasympathetic inhibition as CLZ (Agelink et al., 2001). This was suggested to be the result of CLZ's extreme cholinergic receptor inhibition coupled with antagonism of the α-adrenoceptors. This was supported by another case report of a treatment-resistant patient with schizophrenia who developed tachycardia and reduced HRV after commencing CLZ treatment, with increased LF and decreased HF thereby indicative of increased sympathetic control (Cohen et al., 2001b). The cardiovascular signs subsided after switching to OLA.

Pulse rate variability (PRV) was further investigated in a study comparing CLZ (*n* = 10) and OLA (*n* = 18). The authors had previously found that PRV yielded results comparable to HRV, with parameters calculated from finger pulses instead of electrocardiograms for the PRV (Mueck-Weymann et al., 2002). CLZ-treated patients displayed significantly increased HR and diminished HRV parameters compared to healthy controls. OLA-treated patients displayed the same trend, albeit to a much lesser extent (Mueck-Weymann et al., 2002). The authors attribute the observed autonomic changes to the presence of CLZ in plasma at high levels and CLZ's strong antagonism of cholinergic and adrenergic receptors.

As mentioned above, multiple studies have noted reduced HRV with CLZ use, an observation believed to be due to antagonism of muscarinic receptors in the vagus nerve. Rechlin et al. (1998) sought to expand upon this research by further including measurements of plasma CLZ drug levels in their study. Plasma CLZ levels were found to be inversely correlated with HRV parameters, with the largest decrease in HRV occurring during periods of deep breathing and rest (Rechlin et al., 1998). The authors speculate that HRV parameters are increased in the standing position due to higher sympathetic activity (Rechlin et al., 1998). The authors have previously shown non-medicated patients with schizophrenia have elevated HR and normal HRV compared to healthy controls, whilst CLZ-treated patients had tachycardia and diminished HRV (Rechlin et al., 1994). Based on these results, Rechlin et al. raise the possibility of using HRV to predict plasma CLZ concentration when blood sampling is unavailable, yet sensitivity of this method can be an issue since multiple factors such as age and concomitant medication can affect HRV (1998). Alternatively, measurement of plasma CLZ concentration can be used to determine the threshold of HRV decrement to elicit a therapeutic response, but will require further studies which may prove difficult (i.e., controlled studies with differing doses; Rechlin et al., 1998).

Although the use of HRV in reporting autonomic dysfunction in psychotic patients has become increasingly popular, most of the studies performed have focused on linear components of HRV rather than non-linear measures (Kim et al., 2004). Non-linear measures of HRV take into consideration that HR is part of a dynamic biological system, and although unpredictable in the long run (i.e., in a non-linear manner), the trajectory of the system can be theoretically determined using complex mathematical equations dependent on initial conditions of the system (Mansier et al., 1996). Kim et al. (2004) incorporated non-linear HRV parameters into their assessments of CLZ-induced autonomic dysfunction in patients with schizophrenia, demonstrating non-linear parameters were diminished with CLZ treatment, thereby indicating decreased autonomic control over cardiac function. Of note, the lack of correlation between sample entropy (SampEn) and CLZ dosage suggests psychosis directly influences autonomic function (Kim et al., 2004). SampEn is a complexity and regularity measure of time series data that removes bias by not including self-matches in calculations of probability and is largely independent of data length (Richman and Moorman, 2000). SampEn is inversely correlated with regularity, where higher values reflect randomness in the time series of HR recordings and thus arrhythmia (Kim et al., 2004). The authors propose to include non-linear HRV parameters to associate symptom severity with cardiac function.

Another study connecting psychosis symptom severity with cardiac function was conducted by Bär et al. (2005) in paranoid patients with schizophrenia, with HRV assessments taken before and after neuroleptic treatment. It was shown non-medicated patients displayed tachycardia and decreased HRV with no change after initiating antipsychotic treatment, suggesting schizophrenia directly affects autonomic activity (Bär et al., 2005). However, the study included a range of antipsychotics at inconsistent dosing regimens and will need to be replicated in a larger sample population.

The sole HRV preclinical study identified in our literature search was conducted by Wang et al. (2012), on male Wistar-Kyoto rats. This study also took into consideration the influence of antipsychotics on sleeping patterns and consequently autonomic activity during sleep-wake cycles. Results show rats treated with CLZ displayed accelerated HR and decreased RR, TP, HF, and LF%. When sleep-wake cycles were accounted for, the CLZ group demonstrated longer periods of wakefulness, to which the magnitude of the RR decrease was found to be less than that during sleep, implying CLZ-induced tachycardia was more severe during sleep (Wang et al., 2012). Of note, only animals treated with CLZ displayed significantly different RR, TP, and LF% values compared to controls regardless of consciousness. HAL and risperidone did not significantly influence HRV in the same manner as CLZ, which the authors attributed to CLZ's strong anticholinergic and antiadrenergic properties (Wang et al., 2012).

### Postural hypotension and respiratory sinus arrhythmia

Several non-invasive cardiovascular tests have been developed to identify abnormalities in the ANS. These include recording HR response to the Valsalva maneuver, postural change and deep breathing, and BP response to sustained handgrip and postural change. Although these conventional tests are not as informative as spectral analysis of HRV, they are still used due to ease of implementation and they offer early detection of autonomic dysfunction when continuous ECG monitoring is unfeasible (Ewing and Clarke, 1986).

HR changes in response to posture and deep breathing reflect both sympathetic and parasympathetic reflex activity. HR is expected to increase rapidly when the subject moves from a supine to a standing position, then decreases as reflex bradycardia ensues. The characteristic increase in HR will be compromised in diseased states, such as diabetes (Ewing and Clarke, 1986). In a similar test, BP is first recorded while the subject is in a supine position and repeated when the subject is standing. The difference in systolic BP (SBP) in the supine position and SBP when standing reflects postural fall in SBP, which normally should be <10 mmHg since sympathetic vasoconstriction rapidly restores SBP (Ewing and Clarke, 1982).

Testing HR response during deep breathing involves asking the subject to breathe deeply at a constant rate and calculating the differences between the maximal and minimal HRs during each cycle. Autonomic neuropathy will abolish the HRV that usually varies in sync with breathing cycles (Ewing and Clarke, 1982).

The Valsalva maneuver requires the seated subject to maintain a mercury manometer at 40 mmHg for 15 s by blowing into a mouthpiece. The results are expressed as ratios, where the longest RR interval in the 20 heart beats immediately after the maneuver is divided by the shortest RR interval during the maneuver. The maneuver is intended to induce stress where a drop in BP is accompanied by HR elevation. In healthy individuals, there is a reflexive overshooting of BP above baseline values and decreasing of HR after stress. Compromised parasympathetic regulation prevents BP from overshooting and HR remains unchanged following stress (Ewing and Clarke, 1982).

BP response during sustained handgrip evaluates sympathetic function, where sustained isometric muscular contraction increases cardiac output and results in a sharp increase in BP (Ewing and Clarke, 1982). The subject's maximum grip on a dynamometer is recorded and 30% of the recorded power is maintained over 5 min. Results are expressed in terms of diastolic BP (DBP) differences, where the difference between resting DBP is subtracted from the highest DBP during sustained handgrip. DBP should rise rapidly when commencing the handgrip from increased cardiac output, where individuals with autonomic neuropathy will show minimal increases in DBP (Ewing and Clarke, 1982).

Nielsen et al. (1988) studied the influence of postural change and breathing on HR in medicated and non-medicated patients with schizophrenia compared to healthy controls. Antipsychotics taken by the medicated group included flupentixol, CLZ and sulpiride. HR was continuously recorded over 15 min in the supine position and followed by a period of 5 min in the standing position. The difference in HR between the supine and standing positions were used to assess sympathetic activity, where an elevation of HR over 30 bpm was considered abnormal (Nielsen et al., 1988). In the resting supine position, medicated patients with schizophrenia had significantly higher HR than the non-medicated and control groups (Nielsen et al., 1988). However, postural changes resulted in significant HR responses in both medicated and non-medicated patients in comparison to healthy controls.

Testing for changes in HR during stages of respiration consisted of a 5 min resting period with normal breathing, followed by complete exhalation then deep and rapid inspiration. Subjects were asked to hold their breath for 20 s after inhaling and the difference between the maximal and minimal HR immediately after inspiration was used to denote HR response. Abnormal HR response was defined as a difference of <10 bpm compared to the norm of >15 bpm (Nielsen et al., 1988). HR response to deep inspiration was significantly higher in non-medicated patients with schizophrenia compared to both the medicated group and controls. However, all 3 groups had normal HR response ranging from 23 to 31 bpm, with only the medicated group showing any abnormality (3 out of 28 subjects).

In a later study, Agelink et al. (1998) modified the postural testing procedure to 10 min in the supine position preceding immediate standing, with the ratio of the 15th and 30th heartbeat reflecting sympathetic activity (30:15 ratio). The 15th heartbeat is indicative of the maximal increase of HR following the change in posture, whereas the 30th heartbeat represents peak reflex bradycardia (Agelink et al., 1998). Unlike Nielsen et al's. study (1988) where data of several different medications were pooled, Agelink et al. (1998) focused on comparing the individual drug effects of HAL and CLZ on cardiovascular autonomic parameters. A total of 46 inpatients with schizophrenia were recruited, where *n* = 20 received CLZ and *n* = 26 received HAL. The control group consisted of 30 healthy subjects. Resting HR and BP were significantly higher in the CLZ-treated group compared to HAL and controls. In addition, CLZ treatment significantly reduced 30:15 ratios, HR during deep breathing and BP during sustained handgrip, suggestive of autonomic dysfunction.

### Electrodermal activity

Electrodermal activity (EDA) or skin conductance is another widely used measure for sympathetic activity. Arousal of the SNS is directly correlated to sweat gland activity, as measured by electrical conductivity in the skin. There is evidence of both extremes of the EDA spectrum in patients with schizophrenia, known as non-responders (hypoactivity) and responders (hyperactivity), respectively (Ohman, 1981; Dawson and Nuechterlein, 1984; Nilsson et al., 2015). EDA is also indicative of social cognition regulation, as non-responders comprise a subgroup of patients with schizophrenia who display less empathy and are comparable to healthy controls, in contrast to responders who demonstrate significantly higher EDA and self-reported distress (Dawson and Schell, 2002; Schell et al., 2005; Ikezawa et al., 2012). In a study combining EDA and HRV, Zahn and Pickar (1993) demonstrate CLZ induced tachycardia, suppressed HRV and decreased skin conductance responses in comparison to placebo. Of interest, fluphenazine despite possessing anticholinergic properties, did not significantly affect HR to the same extent as CLZ. The authors suggest the discrepancy is due to CLZ being a more potent cholinergic and α_2_-adrenoceptor antagonist than fluphenazine (Zahn and Pickar, 1993).

### Catecholamines

Due to accessibility issues and invasiveness of measuring direct neuronal activity in humans, sampling of the major catecholamines norepinephrine (NE), epinephrine (E), and dopamine in plasma or urine has become increasingly popular for assessing sympathetic activity in health and disease (Esler et al., 1990; Forslund et al., 2002). Elevated plasma NE levels have been linked to a number of cardiovascular anomalies including heart failure (Thomas and Marks, 1978; Cohn et al., 1984; Hasking et al., 1986), hypertension (Rumantir et al., 2000; Masuo et al., 2003; Schlaich et al., 2004), left ventricular hypertrophy (Zoccali et al., 2002; Schlaich et al., 2003) and postural tachycardia (Shannon et al., 2000; Goldstein et al., 2002; Mayer et al., 2006). Importantly, CLZ is known to substantially increase plasma NE spillover compared to conventional antipsychotics (Pickar et al., 1992; Green et al., 1993; Brown et al., 1997; Spivak et al., 1998).

There is evidence of elevated catecholamines associated with the use of CLZ. Li et al. (1997) reported a rise in BP from a low of 110/70 mmHg to a maximum of 146/106 mmHg in a patient during the course of 10 weeks of CLZ treatment. The hypertension was accompanied by rise in urinary E and NE, to which all 3 anomalies subsided after interventions were introduced and CLZ was discontinued (Li et al., 1997). Paroxysmal hypertension was diagnosed, as with multiple other case reports of CLZ-associated hypertension and raised urinary catecholamines (Krentz et al., 2001; Akinsola and Ong, 2011; Sara et al., 2013). In addition to hypertension, Akinsola and Ong (2011) reported tachycardia of 140 bpm in a patient and Krentz et al. (2001) reported 3 cases of accelerated HR ranging from 104 to 130 bpm. The authors postulated CLZ-induced paroxysmal hypertension arose from the increase in NE spillover due to reduced reuptake into sympathetic nerve endings, α-adrenoceptor antagonism and/or increased vesicular fusion (Li et al., 1997; Krentz et al., 2001; Akinsola and Ong, 2011; Sara et al., 2013).

A single-blinded study of in-patients with schizophrenia showed that CLZ-induced tachycardia and orthostatic hypotension were accompanied with a rise in cerebrospinal fluid homovanillic acid (HVA), a product of dopamine metabolism, in the first 5 days of treatment (Gerlach et al., 1974). Subsequently, Breier et al. (1994) investigated the role of catecholamines and their metabolites in a randomized, double-blinded study. Psychiatric outpatients were given either CLZ or HAL, for a period of 10 weeks. Blood samples, BP and HR were taken prior to the initiation of and during the 5th week of treatment. Apart from NE, catecholamine metabolites including 3,4-dihyroxy-_L_-phenylalanine (dopa), dihydroxyphenylacetic acid (DOPAC), 3,4-dihydroxyphenylglycol (DHPG) were analyzed to reflect adrenergic action. A significant surge in plasma NE was evident in the CLZ group, which was statistically correlated with an increase in dopa (Breier et al., 1994). Despite a notable increase in HR, it was not correlated with the change in plasma NE (Breier et al., 1994). Of interest, the change in NE was correlated with positive outcome as indicated by lower scores in the Brief Psychiatric Rating Scale (BPRS) and Simpson-Angus Scale, suggestive of noradrenergic contribution to clinical efficacy (Breier et al., 1994). Due to unchanged DHPG levels, an increase in the intraneuronal metabolism of NE does not explain the observed surge in plasma NE. The authors speculate NE transporter (NET) inhibition played a role in increased circulating NE (Breier et al., 1994).

As elevated catecholamines have been linked to myocarditis, Wang et al. (2008) used a mouse model to investigate the effect of CLZ on heart structure and the release of NE, E, and the inflammatory marker TNF-α. CLZ was administered daily to young mice at varying doses, over a period of 7 or 14 days. Histological analysis revealed inflammation of the myocardium peaked on day 7, whereas NE and E were persistently elevated (Wang et al., 2008). Of note, TNF-α was only measured on day 14 and was found to be significantly elevated after CLZ treatment in a dose dependent manner (Wang et al., 2008).

### Interventions

Of the 37 studies in our systematic review, 16 studies have reported using interventions and/or discontinuing CLZ to treat cardiovascular side effects. Commonly occurring side effects and their respective interventions will be discussed below.

### Tachycardia

Tachycardia is defined by a HR above 100 bpm, accompanied by a mean HR of more than 90 bpm within a 24 h period (Sheldon et al., 2015). Accelerated HR is a commonly reported side-effect of CLZ and can precede fatal cardiac myopathies (Jones et al., 2014). Baciewicz et al. (2002) reported a patient who adversely responded to CLZ, with HR elevated to 150 bpm after 3 weeks of treatment. The patient received 5 mg of metoprolol, a β-adrenoceptor blocker, to alleviate the tachycardia (Baciewicz et al., 2002). Hypotension resulted from the metoprolol intervention, but symptoms subsided upon CLZ discontinuation.

Pereira et al. (2010) noted a case of neuroleptic malignant syndrome (NMS) in a 56 year old male patient with schizophrenia, characterized by profuse sweating, hand tremors, tachycardia and rigid limbs resulting from antipsychotic medication. The patient was previously on chronic chlorpromazine (150 mg/day, 10 years), CLZ (200 mg/day, 3 years) and atenolol (50 mg/day, 15 years). The patient's condition worsened as he developed convulsions on the 14th day of hospitalization, to which he was treated with phenytoin, divalproex sodium and bromocriptine. Discontinuation of CLZ resolved the NMS. The authors attribute the NMS to CLZ, since NMS should be apparent within the first month of chlorpromazine treatment while it can still emerge beyond 3 years of CLZ treatment (Pereira et al., 2010). Further evidence is provided by another 2 case reports of patients diagnosed with NMS (Yacoub and Francis, 2006). Both patients were given CLZ at 100–175 mg/day before NMS was diagnosed within 2 weeks of initiating CLZ treatment. Symptoms included unstable BP and HR as well as diaphoresis for one of the patients, which were resolved by withdrawing CLZ and introducing lorazepam.

Sinus tachycardia developed in a patient who attempted suicide by CLZ overdose, accompanied by sedation, hypothermia and hypersalivation (Thomas and Pollak, 2003). The patient ingested 3,500 mg of CLZ, resulting in hospitalization and treatment with naloxone and thiamine for the overdose. Serum drug levels deviated from the known CLZ half-life of 12 h, remaining at therapeutic levels of >300 ng/ml for 6 days instead of declining to negligible levels within 2 days (Thomas and Pollak, 2003). High HR also persisted (110–136 bpm) for 6 days, coinciding with the duration of the elevated serum CLZ levels. The plot of serum concentration versus time was of a biphasic pattern and consistent with a sustained release model, believed to be the result of CLZ accumulation and/or decreased bowel movements in the patient (Thomas and Pollak, 2003). Leo et al. (1996) also report a patient with sinus tachycardia after commencing CLZ at 750 mg/day. The dose was eventually lowered to 700 mg/day for 5 months, until persistent tachycardia and left ventricular (LV) abnormalities arose, to which CLZ was discontinued and digoxin (0.25 mg/day) was prescribed. Digoxin inhibits sodium-potassium ATPase to increase myocardial contractile force, sensitizes baroreceptors and promotes vagal control (Gheorghiade et al., 2004). The patient's cardiac anomalies were attributed to an existing cardiomyopathy exacerbated by CLZ (Leo et al., 1996).

Koren et al. (1997) reported a patient whose conditions deteriorated rapidly despite tolerating CLZ treatment for 11 weeks. The patient developed ventricular tachycardia and was treated unsuccessfully with lidocaine, eventually passing away within 36 h of hospitalization. The authors postulate the fatality was due to agranulocytosis and cardiomyopathy caused by lactic acidosis or myocarditis (Koren et al., 1997). The direct action of CLZ on membrane proteins may play a role in lactic acidosis, specifically CLZ's suppression of calcium-dependent potassium permeability leading to decreased insulin secretion (Koren et al., 1997).

A sixth report is of a young patient with schizophrenia who developed persistent tachycardia as a result of initiating CLZ for 1 week (Ennis and Parker, 1997). HR notably increased from 80 bpm to a maximum of 130 bpm, accompanied by hypertension upon commencement of CLZ.

### Hypertension

Hypertension refers to a SBP above 120 mm Hg and a DBP above 80 mmHg, where individuals with chronically elevated BP above 140/90 mmHg are at a higher risk of developing premature CVD (Giles et al., 2005). In addition to persistent tachycardia that was noted in the previously mentioned case report, Ennis and Parker (1997) also noted hypertension in their patient. The authors ruled out common causes of hypertension, since blood tests, urinary catecholamines and physical examinations showed no obvious abnormalities (Ennis and Parker, 1997). Atenolol, a β_1_-adrenoceptor antagonist, successfully stabilized the BP and HR, suggesting circulating catecholamines play a role in causing the hypertension (Ennis and Parker, 1997).

Li et al. (1997) reported a case of CLZ-induced pseudophaeochromocytoma, with the patient's BP rising up to 146/106 mmHg from 110/70 mmHg. The patient initially received CLZ at a dose of 25 mg/day, which was gradually titrated to 300 mg/day over the course of 10 weeks. Propranolol (PRO) and amlodipine were administered to reverse the hypertension. PRO and amlodipine lowers BP via β_1_-adrenoceptor and calcium channel antagonism, respectively (Li et al., 1997). The patient's hypertension resolved after medication, although this may also be due to withdrawal of CLZ.

Similarly, Prasad and Kennedy (2003) noted hypertension in their patient 5 days after commencing CLZ at 50 mg/day. However, treatment with amlodipine at 5 mg/day was ineffective and the hypertension persisted until CLZ was withdrawn at 13 weeks. The authors postulate CLZ's hypertensive effects likely resulted from pseudophaeochromocytoma and the effects were transient (Prasad and Kennedy, 2003).

### Hypotension

CLZ impeded vasopressor responses in a patient with schizophrenia suffering hemodynamic complications stemming from a subarachnoid hemorrhage (Leung et al., 2015). The patient developed hypotension (mid-90s mmHg) and tachycardia (>120 bpm), despite infusion of phenylephrine, NE, E, and dobutamine. Hypotension is commonly defined as a SBP lower than 90 mmHg, however, 110 mmHg has more recently been suggested (Eastridge et al., 2007). Mean arterial pressure (MAP) was restored following vasopressin administration and temporarily discontinuing CLZ, which removed the influence of CLZ's adrenoceptor antagonism as with the previous vasopressors (Leung et al., 2015).

Another example of CLZ interfering with treatment of hemodynamic disturbances is of a patient with schizophrenia who developed intraoperative hypotension following intubation under anesthesia (John et al., 2010). The patient's MAP decreased from 85 mmHg to 40 mmHg and was unresponsive to E administration. Further decrements in MAP led to the administration of vasopressin, which restored MAP to >65 mmHg. It was suggested CLZ blocked α_1_-adrenergic receptors and caused vasodilation via reflex β_2_-adrenoceptor activation, thereby worsening the already declining MAP (John et al., 2010). Vasopressin successfully induced vasoconstriction since its actions were mediated through the non-adrenergic receptor, V_1_ receptor, thus bypassing the α_1_-adrenergic inhibition. This mechanism perhaps explains the failure of E to raise SBP in the case presented by Koren et al. (1997).

Donnelly and MacLeod (1999) have also presented a clinical case report of a patient on chronic CLZ medication that developed hypotension after a cardiopulmonary bypass procedure. Methoxamine, metaraminol, dopamine and E infusions all failed to maintain the SBP above 60 mmHg, which was eventually restored to >80 mmHg by infusion of NE (Donnelly and MacLeod, 1999). The hypotension was attributed to CLZ-induced vasodilation.

Since melatonin has known benefits in reducing SGA-induced metabolic side effects in animal studies and regulates BP, Romo-Nava et al. (2014) sought to replicate the results in a randomized, double-blinded clinical trial. Patients with schizophrenia (*n* = 24) or bipolar disorder (*n* = 20) were given placebo (*n* = 24) or melatonin (*n* = 20, 5 mg/day) for 8 weeks and anthropometric variables were measured prior to and after the trial. All subjects were previously on SGA medication (CLZ, OLA, risperidone or quetiapine) for ≤3 months before recruitment. Although melatonin lowered DBP in all patients, the decrease was not statistically different from that of the placebo group in patients with schizophrenia. Metabolic measures also did not improve with melatonin use in patients with schizophrenia, while patients with bipolar disorder showed favorable outcome. The lack of therapeutic benefits for the schizophrenia patients is perhaps due to pooling data from 4 SGAs with varying risk for adverse cardiometabolic side effects instead of assessing the neuroleptics individually (Romo-Nava et al., 2014).

## Discussion

This review is a compilation of literature concerning cardiovascular anomalies associated with autonomic dysfunction in CLZ-treated patients with schizophrenia. We have highlighted case reports, clinical trials and animal studies to demonstrate the importance of monitoring cardiovascular parameters when administering CLZ. Patients with schizophrenia inherently have a higher risk for CVD than the general population, due to unhealthy lifestyle habits and use of SGAs (Kelly et al., 2010). Indeed, our results show CLZ is associated with fatal myocarditis, orthostatic hypotension, paradoxical hypertension and tachycardia. The results identified several characteristic features of CLZ's effects on the ANS: increased sympathetic activity, decreased vagal contribution, strong anticholinergic and antiadrenergic properties, increased E and NE and decreased HRV (Thomas and Marks, 1978; Nielsen et al., 1988; Zahn and Pickar, 1993; Ennis and Parker, 1997; Li et al., 1997; Agelink et al., 2001; Cohen et al., 2001a,b; Tümüklü et al., 2008; Wang et al., 2008, 2012; John et al., 2010; Mathewson et al., 2012; Huang et al., 2013; Sara et al., 2013; Leung et al., 2015). The studies, however, are short in duration with the longest clinical trial lasting 6 months (Huang et al., 2013). Side effects such as cardiomyopathy can occur after 12 months, hence future studies need to factor in the time of onset of relevant symptoms (Iqbal et al., 2003). CLZ is also frequently combined with other antipsychotic drugs (Honer et al., 2007, 2009; Procyshyn et al., 2010), even though the evidence indicates that polypharmacy has no therapeutic benefit and may increase metabolic side-effects (Boyda et al., 2010, 2013). It will therefore be important to determine if CLZ's cardiovascular effects are exacerbated by antipsychotic polypharmacy.

Less than half (16 out of 37) of the included studies have used interventions that have successfully attenuated CLZ-induced cardiovascular complications. These interventions include metoprolol, lorazepam, atenolol, PRO, amlodipine, vasopressin and NE infusion (Ennis and Parker, 1997; Li et al., 1997; Donnelly and MacLeod, 1999; Baciewicz et al., 2002; Yacoub and Francis, 2006; John et al., 2010; Leung et al., 2015). The drugs were administered in response to tachycardia, hypotension or paradoxical hypertension in individual case reports. Replication of the beneficial results in randomized, placebo-controlled trials with larger sample sizes is therefore warranted.

In contrast to the numerous reports of cardiovascular anomalies from CLZ use, there is a lack of in-depth investigations on the underlying causes of these adverse events. Breier et al.'s (1994) study of plasma NE metabolites is a rare trial that had explored mechanistic relationships between CLZ's superior efficacy and adrenergic function. A hallmark of CLZ-treated patients is the dramatic elevation of plasma NE levels, caused by inhibition of NE reuptake into postganglionic terminals, increased NE vesicular fusion, downregulation of β-adrenoceptors and/or inhibition of NET. It is widely believed increased sympathetic nerve traffic reflects increased NE spillover and plasma NE levels, but this is not always the case since vesicular leakage is also a major determinant of plasma NE levels as extensively reviewed elsewhere (Giles et al., 2005; Sheldon et al., 2015). Often underappreciated, vesicular leakage of NE into the cytoplasm ensures sustainable release beyond the maximal rate of synthesis by tyrosine hydroxylase activation to keep pace with NE release (Sheldon et al., 2015). Through measuring multiple plasma catecholamine metabolites that served as indicators of NE synthesis, release and reuptake, Breier et al. (1994) showed the surge in plasma NE levels was likely the result of combined inhibition of α-adrenoceptors and NET. A follow-up study from the same group further pinpointed the mechanism to be one of increased NE spillover, explained by increased vesicular fusion at the axonal membrane (Elman et al., 1999). Although these observations are pure speculation without further pharmacological studies, the inclusion of specific intraneuronal indicators of NE turnover and metabolism (i.e., DHPC, dopa, DOPAC) suggest increased NE spillover from vesicular fusion is possible. The studies, however, only document the acute effects of CLZ treatment on adrenergic function. Chronic studies are needed to determine the long-term role of NE spillover in autonomic neuropathy.

Autonomic tests such as changes in BP and HR in response to standing or deep breathing are also indicative of irregularities in the ANS. While test results are commonly used to reflect damage to the SNS and/or the PNS, they are unlikely to be solely parasympathetic or sympathetic in nature and instead represent a complex intertwine of the 2 branches of the ANS (Ewing and Clarke, 1982). Results from the “battery” of autonomic tests should be interpreted with caution as significant differences may not necessarily indicate severe autonomic neuropathy. Agelink et al. (1998) report significant differences in CLZ-treated groups, yet when the actual values are considered, the anomalies are categorized as “borderline” instead of “pathological.” The reported 30:15 ratio, deep breathing HR response and handgrip BP response in CLZ-treated patients in this study all fall under the borderline category (Ewing and Clarke, 1982; Agelink et al., 1998). Nevertheless, autonomic tests remain as important indicators of autonomic dysfunction and are pivotal in early prevention of CLZ-induced CVD.

It is important to note our results yielded only 1 publication concerning myocarditis and cardiomyopathy. Given the grave consequences of myocarditis and cardiomyopathy, further elaboration of our results and pathogenesis of these cardiac disorders are warranted. Firstly, the focus of our review is on autonomic dysfunction associated with CLZ while it is believed CLZ-induced myocarditis arises from type 1 hypersensitivity mediated by immunoglobulin E (IgE) (Kilian et al., 1999) or of viral origin (Merrill et al., 2006). IgE-mediated hypersensitivity is the most convincing and widely accepted hypothesis to date, as the time to emergence of eosinophilic infiltrates corresponds accordingly to the time course of a type I allergic reaction and duration of CLZ treatment (Kilian et al., 1999; Ronaldson et al., 2015). Ensuing cardiomyopathy is believed to be the result of the direct cardiotoxic effects of CLZ or a type III hypersensitivity reaction (Merrill et al., 2005; Ronaldson et al., 2010). While there has been suggestion of catecholamine involvement in myocarditis and subsequent cardiomyopathy (Merrill et al., 2006; Wang et al., 2008), no definitive clinical evidence has been presented thus far. Secondly, underreporting and misdiagnosis of the disorders frequently occur due to the variability of the symptoms associated with myocarditis (Ronaldson et al., 2015). CLZ-induced myocarditis can be overlooked as initial symptoms such as fever and tachycardia closely resemble normal CLZ dose titration and are typically resolved by discontinuation of CLZ before myocarditis can be diagnosed (Merrill et al., 2006; Ronaldson et al., 2015). Fatalities associated with myocarditis can also be missed since diagnosis would require an autopsy and histological assessment of the myocardial region concerned (Ronaldson et al., 2015). Under these circumstances the suspicion for myocarditis is low and would not generally warrant further monitoring and investigation. The correct diagnosis of myocarditis necessitates relevant cardiac monitoring at the time of onset of myocardial damage and awareness from psychiatrists. The abovementioned factors may have contributed to the lack of publications involving myocarditis and cardiomyopathy in our results.

Lastly, we summarize and propose additional measures to the current cardiac monitoring procedures to minimize risk for cardiac complications when commencing CLZ. The first 4 weeks of starting CLZ treatment should incorporate weekly monitoring of inflammatory (C-reactive protein; CRP) and myocardial damage (troponin I or T) markers in routine blood work for signs of myocarditis. As the onset of myocarditis is typically within 14 – 22 days of the start of CLZ treatment, it is critical to closely monitor patients for fever, troponin I or T and CRP levels for the first 28 days of treatment (Ronaldson et al., 2010, 2011). In a systematic review of CLZ-induced myocarditis, Ronaldson et al. noted the onset of fever and other symptoms non-specific for myocarditis after 10–19 days of commencing CLZ (2011). These symptoms should not be ignored especially when accompanied by CRP levels over 50 mg/L, which signify the onset of myocarditis (Ronaldson et al., 2011). Vital signs including BP, body temperature, HR and respiratory rate should be recorded every second day. Elevations in HR above 20–30 bpm can occur with the onset of initial symptoms and precede rising troponin I/T levels. In the event of CRP levels rising above 100 mg/L or troponin levels greater than 2 ULN, CLZ should be immediately discontinued, as these 2 measures represent the best sensitivity for symptomatic cases (Ronaldson et al., 2011; Freudenreich, 2015). Baseline echocardiography and weekly ECGs are ideal, but the former is unnecessary for identifying pre-existing cardiac complications and the latter lacks sensitivity in detecting myocarditis (Stein et al., 1994; Ronaldson et al., 2011). Since there is concern for exhausting available health care resources, it has been suggested that an ECG is required only when myocarditis is suspected (Freudenreich, 2015). However, we recommend the inclusion of a weekly ECG to detect abnormal changes in HRV and performing weekly autonomic tests during the critical first 4 weeks of CLZ treatment. These measures can be incorporated with the mandatory blood sampling each week to reduce any burden on health care resources. Early detection of cardiac abnormalities is not only crucial for preventing fatalities, but will also reduce the eventual costs of health care in the long run. Every effort should be made in maximizing the therapeutic benefits of CLZ, which remains heavily underutilized in managing treatment-resistant schizophrenia (Kar et al., 2016).

## Conclusion

Reduced HRV, elevated catecholamines, tachycardia and hypotension are known effects of CLZ treatment. Yet there is a lack of controlled trials to confirm that these autonomic abnormalities are caused specifically by CLZ. Future studies should incorporate both mechanistic relationships as well as interventions to minimize CLZ's adverse effects on cardiovascular function, such as different dosing strategies (Procyshyn et al., 2014). Moreover, CLZ's strong antagonistic properties on multiple receptors should be considered before prescribing CLZ to patients with cardiovascular complications, as evidenced by failure to restore cardiac homeostasis in several patients using conventional adrenergic agonists. Clinicians should consider the use of HRV and conventional autonomic tests to preliminary screen for patients with a higher risk for cardiovascular adverse events before commencing CLZ treatment and to continuously monitor cardiovascular function once CLZ is prescribed.

## Author contributions

JY performed the systematic search and wrote the manuscript, in collaboration with AB. All other authors assisted with the review and contributed to the final draft of the manuscript.

### Conflict of interest statement

AB has received grants from Bristol-Myers Squibb. WH has received consulting fees or sat on paid advisory boards for *in silico*, Lundbeck, Otsuka, Roche, and Eli Lilly, and received honoraria from Rush University, University of Calgary, University of Hong Kong, Massachusetts General Hospital, British Columbia Health Authorities, the British Association for Psychopharmacology and the Canadian Psychiatric Association. RP has been a member of the following advisory boards in the past 3 years: Janssen, Lundbeck, and Otsuka. The other authors declare that the research was conducted in the absence of any commercial or financial relationships that could be construed as a potential conflict of interest.
