# Supplementary material for: Clozapine-Induced Cardiovascular Side Effects and Autonomic Dysfunction: A Systematic Review
Source: Front Neurosci. 2018 Apr 4;12:203. doi: 10.3389/fnins.2018.00203 (PMC5893810; doi:10.3389/fnins.2018.00203)
Supplement: Supplementary file 1 [file Table1.docx]

**Table 1.** Summary of literature search of clozapine-induced cardiovascular side effects and changes in autonomic function.

| Study type | Author | Subjects, mean age ± SD | Study design | Dose and duration of clozapine | Intervention | Primary outcome | Results | Possible mechanism (s) for autonomic effects | Limitations |
| --- | --- | --- | --- | --- | --- | --- | --- | --- | --- |
| Animal study | Krisch *et al.* (1994) | Female and male Wistar rats (180 – 300 g), male Han: NMRI mice (18 – 25 g) | Assessment of sedative and hypotensive side effects, and the binding profiles of LEK-8829 and LEK-8841 in comparison to CLZ and HAL | 16 – 64 mg/kg | None | Drugs’ K_i_ and pK_i_ for D_1_, D_2_ and 5-HT_2_ receptors, cataleptogenic and locomotor activity, cage-climbing behavior, head-twitch response, ED_50_, sleeping time from pentobarbital injection, muscular tremors from oxotremorine, MAP | All drugs displayed dose dependent decreases in MAP in anesthetized rats. CLZ had stronger hypotensive effects and stronger apomorphine inhibition compared to HAL. CLZ had largest difference between the ED_50_ that blocked apomorphine-induced locomotor activity (regulated by dopamine receptor activation, indicator of psychosis in rats) and the ED_50_ that induced catalepsy (dopamine receptor inhibition, EPS model in rats). This suggests CLZ has high efficacy with minimal EPS. | CLZ has limited liability for EPS due to strong anticholinergic properties. LEK-8829’s cataleptogenic activity attributed to 5-HT_2_ receptor antagonism. | Higher 5-HT_2_ receptor antagonism but lower affinity to D_1_ and D_2_ receptors don’t explain why LEK-8829 was more prone to induce catalepsy than LEK-8841 |
| Animal study | Wang *et al.* (2008) | Male Balb/C mice, 6 weeks old | Preclinical investigation of whether CLZ induced myocarditis and if plasma CAT and cytokine levels exacerbate the severity. Results compared to saline-treated controls. | 5, 10 or 25 mg/kg, 7 or 14 days (n = 8 for each group) | 2 mg/kg PRO | Inflammation grading of H&E stained heart sections, plasma CAT concentration (E and NE), TNF-α | Increased inflammation in CLZ-treated mice. Significant increase in E and NE levels for all CLZ doses compared to saline controls. TNF-α levels significantly increased in 10 and 25 mg/kg CLZ-treated mice. Pre-treatment with PRO reversed the inflammation and elevated CAT and TNF-α levels at the highest dose of CLZ. | Elevated catecholamines contribute to myocarditis severity up to day 7. Downregulation of β-adrenoceptors prevent further adverse effects from excessive CAT exposure. Other proinflammatory factors are likely present since PRO didn’t fully restore TNF-α levels to control levels | Only 1 inflammatory marker was analyzed. Did not include PRO groups for day 7 and for the other 2 doses of CLZ. |
| Animal study | Wang *et al.* (2012) | Adult male Wistar-Kyoto rats (200 – 250, n = 29) | Preclinical study of autonomic function in rats treated with haloperidol, risperidone or clozapine | 20 mg/kg/day, i.p. (n = 7), 5 days | None | EEG, ECG, EMG, power spectral analysis for sleeping states (AW, QS, PS), HRV analysis | Significantly increased HR in the clozapine group, compared to baseline. Parasympathetic and sympathetic activity as indicated by HF and LF%, respectively, both decreased significantly after clozapine treatment. These observations were present in both awake and sleeping states. | Cardiovascular effects due to anticholinergic and adrenoceptor blocking properties of CLZ. | EEG signaling may be affected by use of antipsychotics |
| Case report | Baciewicz *et al.* (2002) | Male, 35 years old | Case report | 100 – 150 mg/day, 3 weeks | Metoprolol, 5 mg (i.v.), discontinued CLZ | HR | Tachycardia up to 150 bpm, hallucinations, fever, symptoms stopped after metoprolol and discontinuing CLZ | NMS caused by aberrant neurotransmitter levels and altered receptor sensitivity | Single report of an adverse response to CLZ, no comparisons to controls |
| Case report | Cohen *et al.* (2001a) | Male, 30 years old | Case report | 500 mg/day, at least 2 years | Discontinued CLZ | ECG: HR, T-waves, QTc interval, PR and QRS intervals | Sinus tachycardia 130 bpm, increased LF ECG components, decreased HRV and decreased HF ECG components. HR improved and symptoms resolved upon discontinuing CLZ and switching to OLA. | Anticholinergic properties of CLZ | Single report of CLZ-induced changes in cardiac parameters, unclear duration of CLZ, no comparisons to controls |
| Case report | Ennis and Parker (1997) | Male, 19 years old | Case report | 175 mg/day, 7 days | Atenolol | HR, BP, full blood count, serum electrolytes, serum C-reactive proteins, liver, thyroid and renal function tests, urinary catecholamines, chest x-ray, ECG | HR rose from 80 bpm to 120 – 130 bpm after CLZ treatment. SBP rose to 140 – 170 mmHg and DBP was 90 – 115 mmHg from an initial 130/90 mmHg. Unremarkable results from other tests apart from a minor elevation in alanine aminotransferase (56 IU/L). | Increased circulating NE | Lacked direct confirmation that the hypertension was due to CLZ, since the drug was not withdrawn and re-introduced |
| Case report | John *et al.* (2010) | Male, 48 years old, admitted for bronchoscopy, mediastinoscopy and a lung biopsy | Case report | 700 mg/day, unspecified duration | E (5 – 10 µg), vasopressin (5 U) | MAP, HR | Severe intraoperative hypotension (MAP 40 mmHg), resistant to E but resolved by vasopressin (MAP 78 mmHg) | E exacerbated hypotension by acting primarily on β_2_-adrenoceptors, in the presence of α_1_-adrenoceptor blockade. Vasopressin raised MAP by acting on V_1_ receptors, independent of CLZ’s α_1_-adrenoceptor inhibition. | Single report of adverse intraoperative hypotension, unspecified duration of CLZ treatment |
| Case report | Leung *et al.* (2015) | Male, 54 years old, subarachnoid hemorrhage | Case report | Unspecified dosage and duration before admission, temporarily discontinued during hospitalization | Verapamil, 0.9% sodium chloride, phenylephrine (0.4 mg/kg/min), NE (0.2 – 0.5 mg/kg/min), dobutamine (7.5 mg/kg/min), E (0.1 – 0.15 mg/kg/min), vasopressin (0.04 units/min) | HR, BP, CT scan, catheter angiography | Persistent tachycardia >120 bpm, hypotension (MAP <90 mmHg) | α_1_-adrenoceptor antagonism hampered hypertensive effects of phenylephrine and NE. E activated peripheral β_2_-adrenoceptors and decreased MAP. Vasopressin does not act on α and β receptors hence increased MAP. | Unknown dosage and duration of CLZ treatment, use of verapamil, propofol and dexmedetomidine that may contribute to hypotension |
| Case report | Li *et al.* (1997) | Male, 27 years old | Case report | 25 – 300 mg/day, 10 weeks | PRO (30 mg/day), amlodipine (5 mg/day) | BP, urinary E and NE levels, CT scan of adrenal glands and I^131^ MIBG scintigraphy | Hypertension (146/106 mmHg), significant increase in E and NE. Symptoms resolved after discontinuing CLZ. | CLZ inhibits reuptake of NE into postganglionic terminals and increases plasma NE, hence raising BP. | Unknown whether patient responded to CLZ, unknown when PRO and amlodipine were introduced and if the hypertension was resolved due to their introduction or CLZ withdrawal, no data on plasma CAT levels. |
| Case report | Pereira *et al.* (2010) | Male, 56 years old | Case report | 200 mg/day, 3 years | Discontinuation of CLZ and chlorpromazine, lorazepam 4 mg/day, bromocriptine 5 – 22.5 mg/day, phenytoin 300 mg/day, divalproex sodium 500 mg/day, amisulpride 100 – 400 mg/day | Temperature, creatinine phosphokinase (CPK) levels, BP, HR, power and movement, leucocyte count, hemoglobin levels, ESR, renal and liver function tests, serum electrolytes, CSF analysis | Symptoms reminiscent of NMS hyperthermia, tachycardia, tremors, rigidity, hypotension, increased CPK levels, decreased hemoglobin levels. Symptoms resolved after amisulpride and divalproex sodium treatment. | NMS possibly due to 5-HT_1Aa_HT1a receptor hyperactivation, which in turn is due to CLZ inhibition of 5-HT_2Aa_HT2a receptors | Patient was taking chlorpromazine in addition to CLZ |
| Case report | Prasad and Kennedy (2003) | Male, 44 years old | Case report | 50 – 300 mg/day, 13 weeks | Amlodipine 5 mg/day, discontinuation of CLZ | BP, HR, urine catecholamines (NE, E, dopamine), abdominal ultrasound | SBP and DBP rose from an initial 120/70 mmHg to 150/110 mmHg after 5 days of CLZ treatment. HR rose up to 106 bpm. Hypertension persisted until CLZ was discontinued at 13 weeks. Urinary NE (1533 nmol/24 hours) exceeded the reference range at 6 weeks (50 – 900 nmol/24 hours). Urinary NE was lowered to 388 nmol/24 hours 6 weeks after withdrawing CLZ. | Hypertension and elevated urinary NE attributed to pseudophaeochromocytoma syndrome from CLZ treatment | Conclusions based on plasma catecholamines, yet only urinary catecholamines were measured in this study |
| Case report | Sara *et al.* (2013) | Female, 49 years old | Case report | 100 mg/day, unknown duration | 1 mg dexamethasone | BP, urinary-free cortisol and catecholamines (dopamine, E, NE), urea, electrolytes, liver function tests, calcium levels, MRI, MIBG scan | Hypertension (150/99 mmHg), elevated urinary-free NE (835 nmol), pseudopheochromocytoma | Increased NE spillover into circulation and urine from increased vesicular fusion, hypertension due to α_2_-adrenoceptor blockade by CLZ | Unknown duration of CLZ treatment, lost to follow up for 2 years after initial examination |
| Case report | Thomas and Pollak (2003) | Female, 20 years old | Case report | Unknown duration and dosage, history of poor compliance | Naloxone (2 mg, i.v.), thiamine (100 mg, i.v.), prophylactic heparin (10000 U/day), ranitidine (150 mg/day, i.v.), ceftriaxone (1 g/day, i.v.). cefazolin (3 g/day, i.v.), gentamycin (300 mg/day, i.v.) | HR, BP, GCS, respiratory rate (RR), chest x-ray, ECG, routine blood work, body temperature, serum CLZ and NOR concentrations | GCS = 3/15 on arrival, HR = 110 bpm, BP = 156/73 mmHg, sinus tachycardia from ECG, serum CLZ 5 – 2183 ng/ml over 10 days. CLZ and NOR concentrations exhibited a biphasic pattern, with the second decline occurring 6 days post-ingestion. | Blockade of α_1,2_-adrenoceptors induce hypotension. Blockade of M­_1_ receptors decreases bowel movement. Biphasic drug concentrations explained by decreased gut motility and hence delayed absorption. | Exact amount of CLZ and/or other drugs consumed unknown |
| Case report | Yacoub and Francis (2006) | Case 1: irrelevant, Case 2: Male, 62 years old; Case 3: Male, 43 years old | Case reports | Case 2: 100 mg/day, < 6 days; Case 3: 175 mg/day, 14 days | Case 2: CLZ withdrawn, lorazepam (2 – 6 mg/day), quetiapine (200 mg/day); Case 3: CLZ withdrawn, lorazepam (2 – 8 mg/day, 9 days), divaloproex sodium (2000 mg/day) | Temperature, BP, CT scan, CPK, WBC, NMS rating scale score | Both patients had fever (38.1 – 38.3°C), muscle rigidity, CPK 312 – 412 U/L, WBC 7.6 – 12.8 K/L, NMS rating scale score 27. Case 2 had fluctuating BP and HR. Symptoms resolved after discontinuing CLZ and lorazepam was introduced. | Did not discuss | Small sample size, data available for 1 week only, no discussion of possible mechanisms |
| Case report | Donnelly and MacLeod (1999) | Male, 51 years old | Case report | 600 mg/day, unknown duration | Methoxamine (8 mg), metaraminol (2 mg), dopamine (10 – 20 µg/kg/min), E (0.25 µg/kg/min), NE (0.25 µg/kg/min), protamine (250 mg) | BP, pulmonary artery occlusion, central venous pressure, cardiac index, systemic vascular resistance | Arterial hypotension with SBP at 50 mmHg following cardiac surgery. Hypotension reversed by NE infusion. | Hypotension due to CLZ-induced vasodilatation | Single case, CLZ was not stopped to confirm its involvement with the hypotension |
| Case report | Akinsola and Ong (2011) | Female, 51 years old | Case report | 925 mg, unknown daily dosage, 8 years | None | BP, HR, serum CLZ levels, urinary catecholamine levels (NE and E) | Hypertension (250/120 mmHg), tachycardia (140 bpm), serum CLZ levels >1110 µg/L, elevated urinary NE (1402 nmol/d) and E (146 nmol/L). Serum normetanephrine was also above the reference range, at 1940 pmol/L. Pseudophaeochromocytoma was diagnosed. | Pseudophaeochromocytoma caused by increased CLZ levels, which exacerbated existing elevations of serum catecholamines | Single case, concomitant medication included lamotrigine, mirtazapine, zopiclone,  and enalapril |
| Case report | Krentz *et al*. (2001) | Patients with CLZ-induced pseudophaeochromocytoma syndrome (n = 4). Cases 1, 3 and 4 were males aged 27, 38 and 22 years, respectively. Case 2 was a female aged 28 years. | Case reports report | Case 1: 400 mg/day, 2 months; Case 2: 700 mg/day, 12 months; Case 3: 900 mg/day, 18 months; Case 4: 600 mg/day, 3 months | None | HR, BP, BMI, urinary catecholamines (NE, vanillylmandelic acid, normetanephrine) | Elevated urinary catecholamine concentrations in all cases. Tachycardia in 3 cases, ranging from 104 – 130 bpm (HR data unavailable for case 4). All cases were hypertensive (SBP: 143 – 180 mmHg, DBP: 100 – 120 mmHg). Withdrawal of CLZ in cases 1 and 2 lowered the catecholamine concentrations and resolved symptoms. Case 4’s hypertension resolved without discontinuing CLZ. | Increased plasma NE via α_2_-adrenoceptor blockade. Intake of sulpiride in cases 2 and 4 possibly exacerbated symptoms by further blocking presynaptic α_2_-adrenoceptors. | NE levels possibly influenced by concurrent medication: fluoxetine and venlafaxine increase synaptic NE (cases 1 and 3) and paroxetine inhibits reuptake of NE (case 4) |
| Case report | Leo *et al.* (1996) | Female, 26 years old | Case report | Titrated up to 750 mg/day over 4 weeks, then to 700 mg/day for 5 months | Warfarin, furosemide, enalapril, and discontinued CLZ. Digoxin (0.25 mg/day) and thiothixene (30 mg/day) | BP, HR, serial CPK, ECG, cardiac ventriculogram | Reduced BP, weakness and nausea on initiation of CLZ treatment. Sinus tachycardia and ventriculogram showed abnormalities in LV movement and ejection fraction. | Authors propose CLZ exacerbated existing cardiomyopathy since patient still had abnormalities in her ventriculogram after CLZ discontinuation. | Patient noncompliant with medication, lost to follow-up |
| Case report | Koren *et al.* (1997) | Male, 37 years old | Case report | Titrated up to 25 mg/day every 3 days, 11 weeks | E, sodium bicarbonate, insulin, lidocaine | WBC counts, body temperature, BP, HR, respiratory rate, ECG, blood glucose, lactic acid levels, arterial blood gas, transthoracic echocardiography | Agranulocytosis, comatose, hypotension (SBP = 70 mmHg), ventricular tachycardia, severe hyperglycemia, cardiac arrest, lactic acidosis. Patient passed away 36 hours after hospitalization. | CLZ inhibited calcium-dependent potassium channels that decreased insulin secretion, leading to elevated blood glucose and lactic acidosis. The latter may have contributed to myocardial failure. | Unknown cause of death and whether it was caused solely by CLZ |
| Clinical trial | Agelink *et al.* (1998) | In-patients with schizophrenia (n = 46) at mean age of 34.9 ± 9.3 years; healthy controls (n =30) at mean age of 35.4 ± 9.0 years | Prospective trial, patients with schizophrenia were treated with either HAL (n = 26) or CLZ (n = 20) | 75 – 400 mg/day, 4 weeks | None | BP and HR at rest and during autonomic function tests: (30:15 ratio, DBT, Valsalva, sustained handgrip and Schellong) | CLZ-treated patients had significant increases in resting HR and BP and reduced HR variation during autonomic function tests. HAL-treated patients had significantly increased resting HR and a decreased 30:15 ratio. | Increased sympathetic activity | No placebo group, 4 week study, no concurrent plasma sampling of drugs and catecholamines |
| Clinical trial | Cohen *et al.* (2001b) | Patients with schizophrenia aged 21-52 years (n = 56), healthy controls aged 21-50 years (n = 30) | Preliminary trial for HRV assessment, patients were treated with CLZ (n = 21), HAL (n = 18) or OLA (n = 17) | 300 – 700 mg/day, unspecified duration | None | ECG data: mean R-R interval, HR, LF and HF bands, QRS complexes | CLZ-treated patients had significantly higher HR and reduced HRV | Increased sympathetic activity and decreased parasympathetic activity, blockade of sodium/potassium and calcium channels thereby affecting ventricular repolarisation | Not randomized, unknown duration of antipsychotic treatment |
| Clinical trial | Gerlach *et al.* (1974) | Male patients with schizophrenia (n =20), aged 18 – 70 years | Single-blind, cross-over study. Patients randomly treated with CLZ or HAL for 82 days, then switched to the other drug for another 82 days. Wash-out periods of 12-20 days preceded each treatment. | 50 – 800 mg/day, 82 days | None | BPRS, HR, BP, global assessment for side effects, laboratory tests on liver function, ECG, CSF HVA (n = 5) | CLZ was more efficacious in improving BPRS compared to HAL. CLZ significantly increased heart rate and caused transient orthostatic hypotension. | Not addressed | No controls, HVA sample size was too small for statistical analysis. Possible residual effects on receptor sensitivity despite wash-out period. |
| Clinical trial | Huang *et al.* (2013) | Patients with schizophrenia (n = 55) were divided into high (n = 28) or low muscarinic affinity groups (n = 27) depending on current antipsychotic treatment. Mean ages were 45.22 ± 9.78 years for HMA and 44.44 ± 9.42 years for LMA | HRV data was compared between HMA and LMA groups | 200 – 300 mg/day, 6 months, n = 4 | None | BMI, BP, exercise levels, PANSS, HRV: LF, HF, TP, LF% and LF/HF | HRV parameters significantly lower in the HMA group. HMA dose and HF was significantly correlated. Concurrent anticholinergic medication found to be positively correlated with the LMA group. | Lower HRV parameters in HMA group explained by vagal suppression, parasympathetic dysfunction (preganglionic neuronal inhibition) and sympathetic inhibition (α-adrenoceptor blockade) | Small sample number for CLZ group, did not exclude concurrent medications that may affect HRV |
| Clinical trial | Mathewson *et al.* (2012) | Outpatients with schizophrenia (n = 42) with mean age 42.1 ± 6.4 years, age-matched healthy controls (n = 28) with mean age 39.6 ± 8.0 years | Cross-sectional study on the correlation between executive and autonomic functions in patients with schizophrenia | Unspecified dosage and duration, n=13 on CLZ | None | WCST (completed only by patients), heart period, EEG, ECG, spectral analysis to identify respiratory sinus arrhythmia (RSA) | CLZ subgroup performed significantly worse during the WCST and had reduced autonomic control compared to other patients. WCST performance positively correlated with parasympathetic control. | Increased sympathetic activity led to shorter HP and increased resting HR. Decreased parasympathetic control reduced resting RSA. | Small CLZ subgroup with unknown duration and dosage, non-randomized and non-blinded study, WCST results were compared to those published in literature and not study controls, relied on resting HR and RSA to reflect autonomic function. Fatigue may influence results during WCST. |
| Clinical trial | Nielsen *et al.* (1988) | Patients with schizophrenia (n = 28) with mean age 29 ± 6 years, drug-free patients with schizophrenia (n = 10) with mean age 25 ± 5 years, healthy controls (n = 12) with mean age 32 ± 7 years | Comparison of cardiovascular parameters at rest and standing between patients with schizophrenia and healthy controls | Unknown duration and dosage | None | ECG (HR, R-R intervals), BP, MBP = DBP + 1/3(SBP – DBP) | Significantly higher resting HR in patients with schizophrenia receiving antipsychotic treatment. Non-medicated patients with schizophrenia displayed significantly elevated HR response to standing and inspiration as compared to medicated patients with schizophrenia. | Elevated HR response to standing indicative of increased sympathetic activity. Increased HR response during deep inspiration indicates increased parasympathetic activity. Medicated group’s higher resting HRs due to anticholinergic effects. | Treatment groups varied in size, details on antipsychotic medication (duration, dosage, n) missing, relied on 2 tests (standing and inspiration) to reflect sympathetic and parasympathetic function. |
| Clinical trial | Oyewumi *et al.* (2004) | Patients with schizophrenia (n = 37), n = 7 patients lost to follow up upon hospital discharge at 4 weeks | Prospective longitudinal study | Average 97.3 – 486.2 mg/day, 8 weeks | None | Complete blood count, serum clozapine and norclozapine norclozpaine drug levels, BP, HR, temperature | CLZ dose and plasma NOR levels are inversely related to body temperature. Resting and standing SBP significantly related to the NOR to CLZ concentration ratio. | 78% of patients were smokers. Smoking may have promoted CLZ metabolism via activating CYP1A2 enzymes. | Did not exclude smokers, missing data for patients discharged from hospital |
| Clinical trial | Romo-Nava *et al.* (2014) | Placebo-treated patients with schizophrenia (n = 24) with mean age 28.6 ± (9.0) years) and melatonin-treated patients (n = 20) with mean age 30.6 ± (7.5 years) | Randomized, double-blinded clinical trial. Comparison of metabolic indices and psychotic symptoms between patients treated with melatonin (5 mg/day) and placebo. | Mean antipsychotic dose 275.1 mg/day (n = 2), unknown dose for CLZ specifically. Treatment duration was ≤3 months upon study inclusion. | Melatonin (5 mg/day), 8 weeks | Fasting blood, body weight, height, BMI, fat mass percentage, fat mass, lean mass, total body water, hip and waist circumferences, waist:hip , BP, PANSS, CGI-S scale, HDRS, YMRS scale (bipolar disorder patients), CDSS (schizophrenia patients) | Cholesterol levels significantly higher in melatonin-treated patients, with comparable triglyceride levels to controls. Significant decrease in mean DBP in melatonin-treated patients. | Antipsychotic-induced metabolic effects arise from central dysregulation of metabolism by the hypothalamus. Beneficial effects of melatonin limited to patients with bipolar disorder due to restoration of the circadian rhythm. Circadian rhythm remains damaged patients with schizophrenia. | Small sample size, did not limit the variety of antipsychotics and concurrent medications used |
| Clinical trial | Tümüklü *et al.* (2008) | Patients with schizophrenia (n = 16), aged 36.4 ± 11.5 years | Evaluation of arrhythmia in patients prior to and after CLZ treatment | 12.5 – 600 mg/day, 10 weeks | None | PANSS, HRV (HRV mean, SDNN, LF/HF), QTc, QTd, late potentials (fQRS, HFLA, RMS-40), UKU scale | Dose dependent reduction in mean HRV and significant decrease in LF/HF | Antagonism of muscarinic and α-adrenoceptors. | Small sample size, gender effects on HRV may be skewed due to large difference in male (n = 5) and female (n = 11) patient numbers , measurements made only at baseline and at 10 weeks |
| Clinical trial | Zahn and Pickar (1993) | Patients with chronic schizophrenia aged 31.9 ± 7.0 years (n = 25) | Single-blinded, clinical study comparing autonomic effects of CLZ with a conventional antipsychotic and placebo. Treatments consisted of fluphenazine/thioridazine and/or placebo and CLZ, in order. | 444 ± 189 mg/day, >6 weeks | None | Skin conductance and temperature, HR, respiration, finger pulse volume, RT. Measurements taken during rest, tone cues and RT tasks. | CLZ increased resting HR and decreased HRV, reduction in electrodermal activity, in comparison to fluphenazine and placebo | Reduced skin conductance explained by CLZ’s anticholinergic and antihistaminic effects, vasomotor orientation are centrally regulated, HR is elevated due to α_2_-adrenoceptor antagonism | Treatment regime fixed, benztropine was co-administered in most patients receiving fluphenazine, possible habituation effects |
| Clinical trial | Agelink *et al.* (2001) | In-patients with schizophrenia (n = 51). Mean ages for each group: amisulpride (n = 12) at 30.9 ± 7.7 years, OLA (n = 13) at 35.3 ± 7.0 years, sertinodole (n = 13) at 35.8 ± 9.0 years and CLZ (n = 13) at 32.4 ± 6.4 years. | Prospective clinical study comparing effects of amisulpride, OLA, CLZ and sertindole on cardiac function | 100 mg/day, average 14.1 ± 3.7 days | None | ECG, resting HRV (HR, CV, RMSSD, LF, HF) taken before and after treatment | Significant increase in HR, likely due to parasympathetic influence as indicated by CV, RMSSD and HF. | Anticholinergic and antiadrenergic effects of CLZ increased sympathetic activity while decreasing parasympathetic activity to increase HR. | Non-randomized study, didn’t compare to placebo, short treatment duration |
| Clinical trial | Rechlin *et al.* (1998) | Schizophrenia patients (n = 40) aged 40.7 years, healthy age-matched controls (n = 40) aged 41.2 years. | Evaluation of the use of HRV to estimate plasma CLZ levels in patients with schizophrenia | 50 – 600 mg/day, ≥8 weeks | None | Plasma CLZ levels, HR, HRV measures (CV, RMSSD, LF, MF, HF) during rest and deep respiration in supine and standing positions | Elevated HR and decreased HRV. HRV was negatively correlated with plasma CLZ levels. | CLZ suppresses HRV, via reducing parasympathetic cardiac activity | Varying clozapine CLZ dose in patients |
| Clinical trial | Mueck-Weymann *et al.* (2002) | Patients with schizophrenia treated with either CLZ (n = 10) or OLA (n = 8). Ages were 29.6 ± 9.5 years and 29.3 ± 8.2 years for the 2 groups, respectively. Healthy controls (n = 10) were aged 28.7 ± 8.3 years. | Cross-sectional study of the effects of CLZ and OLA on pulse rate variability in patients with schizophrenia, compared to healthy controls | 100 – 600 mg/day, ≥4 weeks | None | HR, PRV (RMSSD, pNN50, total power, VLF power, LF power, HF power, LF/HF) | Both CLZ- and OLA-treated groups had significantly higher HR and lower PRV parameters compared to controls. Of the 2 antipsychotics, CLZ had greater effects on HR and PRV compared to controls. | Increased HR due to CLZ’s anticholinergic effects and α_1_-adrenoceptor antagonism. | Non-randomized, placebo not used. Lacking data on PRV and HR prior to treatment. Implausible mean HR reported for olanzapine group (387.9 ± 7.6 bpm). Inconclusive LF/HF data for clozapine group due to low frequency peaks. |
| Clinical trial | Kim *et al.* (2004) | Patients with schizophrenia aged 34.1 ± 7.7 years (n = 50), healthy controls aged 32.7 ± 9.2 years (n = 50) | Cross-sectional study of the correlation between HRV measures and symptom severity in schizophrenia | 50 – 100 mg/day, ≥4 weeks | None | HRV parameters: R-R interval, RMSSD, pNN50, HF, LF, LF/HF, short-term (*α*1) and intermediate-term (*α*2) fractal scaling, slope of power spectrum (*β*), ApEn, SampEn, CSE and CCE | Patients had significantly lower R-R intervals, RMSSD, pNN50, log HF and log LF. *α*2 fractal scaling was significantly higher and ApEn, SampEn, CSE and CCE were significantly lower in patients. SampEN was negatively correlated with PANSS and positive symptoms. | Reduced time and frequency measures attributed to CLZ’s anticholinergic properties and suppression of parasymapthetic activity. | Lack of a placebo group, unknown effect of CLZ drug levels on HRV and severity of psychotic symptoms |
| Clinical trial | Bar *et al.* (2005) | Patients with paranoid schizophrenia (n = 30) aged 34.2 ± 2.3 years and age-matched healthy controls (n = 30) aged 35.5 ± 2.0 years | Comparison of HRV taken prior to and after initiation of antipsychotic treatment: CLZ (n = 2), HAL (n = 3), OLA (n = 9), quetiapine (n = 4), risperidone (n = 10), ziprasidone (n = 2) | 25 – 50 mg, unspecified daily dosage, 2-3 days | None | HRV (HR, RMSSD, R-R intervals, MCR, VLF, LF and HF bands) during rest, deep breathing and in the tilted position, SAPS, SANS | During rest, HR was significantly higher in patients and there was reduced parasympathetic activity as indicated by decreased RMSSD and HF. HR, RMSSD and MCR were significantly increased in patients during the deep breathing test. Maximum and minimum R-R intervals were significantly different in patients during tilt maneuvers. Duration of illness was significantly correlated with resting RMSSD, HF, LF and MCR before medication. SAPS and VLF were positively correlated. | Parasympathethic underactivity, decreased vagal innervation leaves sympathetic stimulation unregulated in the heart | Small sample size, individual drug effects were not considered, no placebo group, non-randomized |
| Clinical trial | Rechlin *et al.* (1994) | Paranoid schizophrenia patients (n = 20) aged 30.1 ± 10.1 years, health controls (n = 20) aged 29.8 ± 8.6 years. Ten patients were given CLZ in monotherapy. | Clinical study of CLZ’s effects on autonomic function before and after treatment, in comparison to non-medicated schizophrenic patients with schizophrenia and healthy controls | 200 – 600 mg/day, 4 weeks | None | HRV parameters during rest, deep breathing and the Valsalva test: HR, coefficient of variation of R-R intervals, RMRSSDRRSSD, MCR, E-I difference, E/I, Valvalsa ratio, posture index, LF, MF, HF | HR increased significantly in response to CLZ treatment (83.9 bpm to 106.5 bpm). CLZ significantly decreased all HRV parameters during rest, deep breathing and postural changes. | CLZ induced cardiovascular autonomic neuropathy possibly through anticholinergic effects | Small sample size, non-randomized, no placebo, unknown duration of illness |
| Clinical trial | Breier *et al.* (1994) | Chronic Patients with chronic schizophrenia (n = 26). CLZ-treated patients were aged 34.7 ± 5.9 years (n = 11) and HAL-treated patients were aged 36.8 ± 9.3 years (n = 15). | Double-blinded, randomized, parallel group comparison of plasma catecholamines and their metabolites in patients treated with either CLZ or HAL. All patients were pre-treated with fluphenazainefluphenzaine (20 mg/day) for 6 weeks. | Titrated up to 400 mg/day by week 4, 10 weeks | None | Plasma catecholamines and their metabolites (dopa, DOPAC, DHPG, NE), plasma ACTH and CORT, BPRS, Simpson-Angus Scale, HR, BP | CLZ significantly increased plasma NE nearly fivefold more than fluphenazine and haloperidol. Dopa was also significantly elevated, with a strong positive correlation to plasma NE levels. CLZ increased HR (from 70.2 ± 11 bpm to 101.8 ± 9 bpm), independent of plasma NE levels. BPRS scores were negatively correlated with plasma NE levels. | Unchanged DHPG despite increased plasma NE suggests the elevation is not due to increased NE release. Blockade of α_1_- and α_2_-adrenoceptors may contribute to improved clinical outcome and stable BP, but not to elevated plasma NE. Increased NE possibly due to blocked reuptake via inhibition of NET. | Cardiovascular parameters and blood assays were performed at baseline and 5 weeks, but not at 10 weeks. Unknown whether the patients had previously received clozapine. |

ACTH: adrenocorticotropin, ApEn: approximate entropy, AW: active waking, BMI: body mass index, BP: blood pressure, BPRS: Brief Psychiatric Rating Scale, CCE: corrected conditional entropy, CDSS: Calgary Depression Scale for Schizophrenia, CGI-S: Clinical Global Impression - Severity, CORT: cortisol, CPK: creatine phosphokinase, CSE: corrected Shannon entropy, CSF: cerebrospinal fluid, CT: computerized tomography, CV: variation coefficient, DBP: diastolic blood pressure, DBT: deep breathing test, DHPG: dihydroxyphenylglycol, dopa: dopamine , DOPAC: dihydroxyphenylacetic acid, DHPG: 3,4-dihydroxyphenylglycol, E: epinephrine, ECG: electrocardiogram, EEG: electroencephalogram, E-I: longest R-R interval during expiration and shortest R-R interval during inspiration, EMG: electromyography, ESR: erythrocyte sedimentation rate, fQRS: filtered QRS duration, GCS: Glasgow Coma Scale, H&E: hematoxylin and eosin, HAL: haloperidol, HDRS: Hamilton Depression Rating Scale, HF: high frequency, HFLA: duration of high frequency components in low amplitude, HMA: high muscarinic affinity, HP: heart period, HR: heart rate, HRV: heart rate variability, HT: serotonin, HVA: homovanillic acid, LF: low frequency, LMA: low muscarinic affinity, LV: left ventricular, M: muscarinic, MAP: mean arterial pressure, MBP: mean blood pressure, MCR: mean circular resultant, MIBG: metaiodobenzylguanidine, MRI: magnetic resonance imaging, NE: norepinephrine, NET: NE transporter, NMS: neuroleptic malignant syndrome, NOR: norclozapine, OLA: olanzapine, PANSS: Positive and Negative Syndrome Scale, PS: paradoxical sleep, PRO: propranolol, PRV: pulse rate variability, pNN50: pairs of adjacent normal-to-normal (NN) intervals between adjacent QRS complexes that differ by more than 50 ms, divided by the total number of NN intervals, QS: quiet sleep, RMS-40: RMS-40: Root mean square voltage in the last 40 ms of the fQRS, RMSSD: root mean square of successive differences, RSA: respiratory sinus arrhythmia, RT: reaction time, SampEn: sample entropy, SAPS: scale for the assessment of positive symptoms, SANS: scale for the assessment of negative symptoms, SBP: systolic blood pressure, SDNN: standard deviations of all R-R intervals, TNF: tumor necrosis factor, TP: total power, UKU: Udvalg for Kliniske Undersøgelser, VLF: very low frequency, WBC: white blood cell, WCST: Wisconsin card sorting test, YMRS: Young Mania Rating Scale

**Table 2.** Common parameters of HRV as defined by the Task Force.

| Domain | Variable | | Units | Physiological significance | Reference values or range |
| --- | --- | --- | --- | --- | --- |
| Time | NN | Normal-to-normal interval | ms | Interval between QRS complexes from sinus node depolarizations |  |
|  | SDNN | Standard deviation of the NN interval | ms | Includes all cyclic components, estimates total HRV | 100 – 141 ms ([Sztajzel, 2004](#_ENREF_139); [Xhyheri et al., 2012](#_ENREF_157)) |
|  | RMSSD | Square root of the mean squared differences of successive NN intervals >50 ms | ms | Estimation of high frequency variation in HR | 27 ms ([Sztajzel, 2004](#_ENREF_139)) |
|  | pNN50 | Number of NN intervals >50 ms divided by total number of NN intervals | % | Significant correlation with HF ([Mueck-Weymann et al., 2002](#_ENREF_100)), measure of parasympathetic activity ([Xhyheri et al., 2012](#_ENREF_157)) | 3 – 9% ([Sztajzel, 2004](#_ENREF_139); [Xhyheri et al., 2012](#_ENREF_157)) |
| Fre**quency** | VLF | Very low frequency range | ms^2^ | Reflects both sympathetic and parasympathetic control in the very low frequency range | 0.003 – 0.04 Hz ([Huang et al., 2013](#_ENREF_59)) |
|  | LF | Low frequency | ms^2^ | Combinatory measure of primarily sympathetic control with debatable parasympathetic input | 0.04 – 0.15 Hz ([Cohen et al., 2001a](#_ENREF_25)) |
|  | HF | High frequency | ms^2^ | Measure of parasympathetic control | 0.15 – 0.40 Hz ([Quintana et al., 2016](#_ENREF_116)) |
|  | LF/HF | Ratio of LF to HF |  | Reflects the balance of sympathetic and parasympathetic control (ratio value positively related to sympathetic activity) | 3.1 ([Huikuri et al., 1994](#_ENREF_60)) |
|  | TP | Total power | ms^2^ | Overall variance of NN intervals | ≤ 0.4 Hz ([1996](#_ENREF_1)) |

**REFERENCES**

(1996). Heart rate variability: standards of measurement, physiological interpretation and clinical use. Task Force of the European Society of Cardiology and the North American Society of Pacing and Electrophysiology. *Circulation* 93(5)**,** 1043-1065.

Agelink, M.W., Majewski, T., Wurthmann, C., Lukas, K., Ullrich, H., Linka, T., et al. (2001). Effects of newer atypical antipsychotics on autonomic neurocardiac function: a comparison between amisulpride, olanzapine, sertindole, and clozapine. *J Clin Psychopharmacol* 21(1)**,** 8-13.

Agelink, M.W., Malessa, R., Kamcili, E., Zeit, T., Lemmer, W., Bertling, R., et al. (1998). Cardiovascular autonomic reactivity in schizophrenics under neuroleptic treatment: A potential predictor of short-term outcome? *Neuropsychobiology* 38(1)**,** 19-24. doi: nps38019 [pii].

Akinsola, O., and Ong, K. (2011). Pseudophaeochromocytoma associated with clozapine therapy: a case report. *Afr J Psychiatry (Johannesbg)* 14(5)**,** 406, 408. doi: <http://dx.doi.org/10.4314/ajpsy.v14i5.9>.

Akselrod, S., Gordon, D., Ubel, F.A., Shannon, D.C., Berger, A.C., and Cohen, R.J. (1981). Power spectrum analysis of heart rate fluctuation: a quantitative probe of beat-to-beat cardiovascular control. *Science* 213(4504)**,** 220-222.

Alvir, J.M., Lieberman, J.A., Safferman, A.Z., Schwimmer, J.L., and Schaaf, J.A. (1993). Clozapine-induced agranulocytosis. Incidence and risk factors in the United States. *The New England journal of medicine* 329(3)**,** 162-167. doi: 10.1056/NEJM199307153290303.

Andreazza, A.C., Barakauskas, V.E., Fazeli, S., Feresten, A., Shao, L., Wei, V., et al. (2015). Effects of haloperidol and clozapine administration on oxidative stress in rat brain, liver and serum. *Neurosci Lett* 591**,** 36-40. doi: 10.1016/j.neulet.2015.02.028.

Ascher-Svanum, H., Zhu, B., Faries, D., Landbloom, R., Swartz, M., and Swanson, J. (2006). Time to discontinuation of atypical versus typical antipsychotics in the naturalistic treatment of schizophrenia. *BMC psychiatry* 6**,** 8. doi: 10.1186/1471-244X-6-8.

Baciewicz, A.M., Chandra, R., and Whelan, P. (2002). Clozapine-associated neuroleptic malignant syndrome. *Annals of internal medicine* 137(5 Part 1)**,** 374.

Bar, K.J., Berger, S., Metzner, M., Boettger, M.K., Schulz, S., Ramachandraiah, C.T., et al. (2010). Autonomic dysfunction in unaffected first-degree relatives of patients suffering from schizophrenia. *Schizophrenia bulletin* 36(5)**,** 1050-1058. doi: sbp024 [pii]

10.1093/schbul/sbp024.

Bar, K.J., Boettger, M.K., Berger, S., Baier, V., Sauer, H., Yeragani, V.K., et al. (2007). Decreased baroreflex sensitivity in acute schizophrenia. *J Appl Physiol (1985)* 102(3)**,** 1051-1056. doi: 00811.2006 [pii]

10.1152/japplphysiol.00811.2006.

Bar, K.J., Letzsch, A., Jochum, T., Wagner, G., Greiner, W., and Sauer, H. (2005). Loss of efferent vagal activity in acute schizophrenia. *J Psychiatr Res* 39(5)**,** 519-527. doi: S0022-3956(05)00004-X [pii]

10.1016/j.jpsychires.2004.12.007.

Barr, A.M., Procyshyn, R.M., Hui, P., Johnson, J.L., and Honer, W.G. (2008). Self-reported motivation to smoke in schizophrenia is related to antipsychotic drug treatment. *Schizophr Res* 100(1-3)**,** 252-260.

Bobes, J., Arango, C., Garcia-Garcia, M., and Rejas, J. (2010). Healthy lifestyle habits and 10-year cardiovascular risk in schizophrenia spectrum disorders: an analysis of the impact of smoking tobacco in the CLAMORS schizophrenia cohort. *Schizophrenia research* 119(1-3)**,** 101-109. doi: 10.1016/j.schres.2010.02.1030.

Boyda, H.N., Procyshyn, R.M., Tse, L., Xu, J., Jin, C.H., Wong, D., et al. (2013). Antipsychotic polypharmacy increases metabolic dysregulation in female rats. *Exp Clin Psychopharmacol* 21(2)**,** 164-171.

Boyda, H.N., Tse, L., Procyshyn, R.M., Wong, D., Wu, T.K., Pang, C.C., et al. (2010). A parametric study of the acute effects of antipsychotic drugs on glucose sensitivity in an animal model. *Prog Neuropsychopharmacol Biol Psychiatry* 34(6)**,** 945-954.

Breier, A., Buchanan, R.W., Waltrip, R.W., 2nd, Listwak, S., Holmes, C., and Goldstein, D.S. (1994). The effect of clozapine on plasma norepinephrine: relationship to clinical efficacy. *Neuropsychopharmacology* 10(1)**,** 1-7. doi: 10.1038/npp.1994.1.

Brown, A.S., Gewirtz, G., Harkavy-Friedman, J., Cooper, T., Brebion, G., Amador, X.F., et al. (1997). Effects of clozapine on plasma catecholamines and relation to treatment response in schizophrenia: a within-subject comparison with haloperidol. *Neuropsychopharmacology : official publication of the American College of Neuropsychopharmacology* 17(5)**,** 317-325. doi: 10.1016/S0893-133X(97)00073-0.

Buckley, N.A., and Sanders, P. (2000). Cardiovascular adverse effects of antipsychotic drugs. *Drug Saf* 23(3)**,** 215-228.

Bymaster, F.P., Calligaro, D.O., Falcone, J.F., Marsh, R.D., Moore, N.A., Tye, N.C., et al. (1996). Radioreceptor binding profile of the atypical antipsychotic olanzapine. *Neuropsychopharmacology : official publication of the American College of Neuropsychopharmacology* 14(2)**,** 87-96. doi: 10.1016/0893-133X(94)00129-N.

Bymaster, F.P., Rasmussen, K., Calligaro, D.O., Nelson, D.L., DeLapp, N.W., Wong, D.T., et al. (1997). In vitro and in vivo biochemistry of olanzapine: a novel, atypical antipsychotic drug. *The Journal of clinical psychiatry* 58 Suppl 10**,** 28-36.

Casey, D.E. (1989). Clozapine: neuroleptic-induced EPS and tardive dyskinesia. *Psychopharmacology (Berl)* 99 Suppl**,** S47-53.

Chang, J.S., Yoo, C.S., Yi, S.H., Hong, K.H., Oh, H.S., Hwang, J.Y., et al. (2009). Differential pattern of heart rate variability in patients with schizophrenia. *Prog Neuropsychopharmacol Biol Psychiatry* 33(6)**,** 991-995. doi: S0278-5846(09)00157-2 [pii]

10.1016/j.pnpbp.2009.05.004.

Citrome, L., McEvoy, J.P., and Saklad, S.R. (2016). A Guide to the Management of Clozapine-Related Tolerability and Safety Concerns. *Clinical schizophrenia & related psychoses*. doi: 10.3371/CSRP.SACI.070816.

Cohen, H., Loewenthal, U., Matar, M., and Kotler, M. (2001a). Association of autonomic dysfunction and clozapine. Heart rate variability and risk for sudden death in patients with schizophrenia on long-term psychotropic medication. *Br J Psychiatry* 179**,** 167-171.

Cohen, H., Loewenthal, U., Matar, M.A., and Kotler, M. (2001b). Reversal of pathologic cardiac parameters after transition from clozapine to olanzapine treatment: a case report. *Clin Neuropharmacol* 24(2)**,** 106-108.

Cohn, J.N., Levine, T.B., Olivari, M.T., Garberg, V., Lura, D., Francis, G.S., et al. (1984). Plasma norepinephrine as a guide to prognosis in patients with chronic congestive heart failure. *The New England journal of medicine* 311(13)**,** 819-823. doi: 10.1056/NEJM198409273111303.

Conley, R.R., Love, R.C., Kelly, D.L., and Bartko, J.J. (1999). Rehospitalization rates of patients recently discharged on a regimen of risperidone or clozapine. *The American journal of psychiatry* 156(6)**,** 863-868. doi: 10.1176/ajp.156.6.863.

Dawson, M.E., and Nuechterlein, K.H. (1984). Psychophysiological dysfunctions in the developmental course of schizophrenic disorders. *Schizophrenia bulletin* 10(2)**,** 204-232.

Dawson, M.E., and Schell, A.M. (2002). What does electrodermal activity tell us about prognosis in the schizophrenia spectrum? *Schizophrenia research* 54(1-2)**,** 87-93.

De Hert, M., Detraux, J., van Winkel, R., Yu, W., and Correll, C.U. (2011). Metabolic and cardiovascular adverse effects associated with antipsychotic drugs. *Nat Rev Endocrinol* 8(2)**,** 114-126.

Dekker, J.M., Crow, R.S., Folsom, A.R., Hannan, P.J., Liao, D., Swenne, C.A., et al. (2000). Low heart rate variability in a 2-minute rhythm strip predicts risk of coronary heart disease and mortality from several causes: the ARIC Study. Atherosclerosis Risk In Communities. *Circulation* 102(11)**,** 1239-1244.

Donnelly, J.G., and MacLeod, A.D. (1999). Hypotension associated with clozapine after cardiopulmonary bypass. *J Cardiothorac Vasc Anesth* 13(5)**,** 597-599. doi: S1053-0770(99)90016-2 [pii].

Eastridge, B.J., Salinas, J., McManus, J.G., Blackburn, L., Bugler, E.M., Cooke, W.H., et al. (2007). Hypotension begins at 110 mm Hg: redefining "hypotension" with data. *J Trauma* 63(2)**,** 291-297; discussion 297-299. doi: 10.1097/TA.0b013e31809ed924.

Elman, I., Goldstein, D.S., Eisenhofer, G., Folio, J., Malhotra, A.K., Adler, C.M., et al. (1999). Mechanism of peripheral noradrenergic stimulation by clozapine. *Neuropsychopharmacology : official publication of the American College of Neuropsychopharmacology* 20(1)**,** 29-34. doi: 10.1016/S0893-133X(98)00047-5.

Ennis, L.M., and Parker, R.M. (1997). Paradoxical hypertension associated with clozapine. *Med J Aust* 166(5)**,** 278.

Esler, M., Jennings, G., Lambert, G., Meredith, I., Horne, M., and Eisenhofer, G. (1990). Overflow of catecholamine neurotransmitters to the circulation: source, fate, and functions. *Physiological reviews* 70(4)**,** 963-985.

Essali, A., Al-Haj Haasan, N., Li, C., and Rathbone, J. (2009). Clozapine versus typical neuroleptic medication for schizophrenia. *The Cochrane database of systematic reviews* (1)**,** CD000059. doi: 10.1002/14651858.CD000059.pub2.

Essock, S.M., Hargreaves, W.A., Covell, N.H., and Goethe, J. (1996). Clozapine's effectiveness for patients in state hospitals: results from a randomized trial. *Psychopharmacology bulletin* 32(4)**,** 683-697.

Ewing, D.J., and Clarke, B.F. (1982). Diagnosis and management of diabetic autonomic neuropathy. *British medical journal* 285(6346)**,** 916-918.

Ewing, D.J., and Clarke, B.F. (1986). Autonomic neuropathy: its diagnosis and prognosis. *Clinics in endocrinology and metabolism* 15(4)**,** 855-888.

Fineschi, V., Neri, M., Riezzo, I., and Turillazzi, E. (2004). Sudden cardiac death due to hypersensitivity myocarditis during clozapine treatment. *International journal of legal medicine* 118(5)**,** 307-309. doi: 10.1007/s00414-004-0464-1.

Forslund, L., Bjorkander, I., Ericson, M., Held, C., Kahan, T., Rehnqvist, N., et al. (2002). Prognostic implications of autonomic function assessed by analyses of catecholamines and heart rate variability in stable angina pectoris. *Heart* 87(5)**,** 415-422.

Fredrikson, D.H., Boyda, H.N., Tse, L., Whitney, Z., Pattison, M.A., Ott, F.J., et al. (2014). Improving metabolic and cardiovascular health at an early psychosis intervention program in vancouver, Canada. *Front Psychiatry* 5**,** 105.

Freudenreich, O. (2015). Clozapine-induced myocarditis: prescribe safely but do prescribe. *Acta psychiatrica Scandinavica* 132(4)**,** 240-241. doi: 10.1111/acps.12425.

Gerlach, J., Koppelhus, P., Helweg, E., and Monrad, A. (1974). Clozapine and haloperidol in a single-blind cross-over trial: therapeutic and biochemical aspects in the treatment of schizophrenia. *Acta Psychiatr Scand* 50(4)**,** 410-424.

Gheorghiade, M., Adams, K.F., Jr., and Colucci, W.S. (2004). Digoxin in the management of cardiovascular disorders. *Circulation* 109(24)**,** 2959-2964. doi: 10.1161/01.CIR.0000132482.95686.87.

Giles, T.D., Berk, B.C., Black, H.R., Cohn, J.N., Kostis, J.B., Izzo, J.L., Jr., et al. (2005). Expanding the definition and classification of hypertension. *J Clin Hypertens (Greenwich)* 7(9)**,** 505-512.

Goldstein, D.S., Holmes, C., Frank, S.M., Dendi, R., Cannon, R.O., 3rd, Sharabi, Y., et al. (2002). Cardiac sympathetic dysautonomia in chronic orthostatic intolerance syndromes. *Circulation* 106(18)**,** 2358-2365.

Green, A.I., Alam, M.Y., Sobieraj, J.T., Pappalardo, K.M., Waternaux, C., Salzman, C., et al. (1993). Clozapine response and plasma catecholamines and their metabolites. *Psychiatry research* 46(2)**,** 139-149.

Haas, S.J., Hill, R., Krum, H., Liew, D., Tonkin, A., Demos, L., et al. (2007). Clozapine-associated myocarditis: a review of 116 cases of suspected myocarditis associated with the use of clozapine in Australia during 1993-2003. *Drug Saf* 30(1)**,** 47-57. doi: 3015 [pii].

Hasking, G.J., Esler, M.D., Jennings, G.L., Burton, D., Johns, J.A., and Korner, P.I. (1986). Norepinephrine spillover to plasma in patients with congestive heart failure: evidence of increased overall and cardiorenal sympathetic nervous activity. *Circulation* 73(4)**,** 615-621.

Henderson, D.C., Cagliero, E., Gray, C., Nasrallah, R.A., Hayden, D.L., Schoenfeld, D.A., et al. (2000). Clozapine, diabetes mellitus, weight gain, and lipid abnormalities: A five-year naturalistic study. *The American journal of psychiatry* 157(6)**,** 975-981. doi: 10.1176/appi.ajp.157.6.975.

Henderson, D.C., Nguyen, D.D., Copeland, P.M., Hayden, D.L., Borba, C.P., Louie, P.M., et al. (2005). Clozapine, diabetes mellitus, hyperlipidemia, and cardiovascular risks and mortality: results of a 10-year naturalistic study. *The Journal of clinical psychiatry* 66(9)**,** 1116-1121.

Hennekens, C.H., Hennekens, A.R., Hollar, D., and Casey, D.E. (2005). Schizophrenia and increased risks of cardiovascular disease. *American heart journal* 150(6)**,** 1115-1121. doi: 10.1016/j.ahj.2005.02.007.

Honer, W.G., Jones, A.A., Thornton, A.E., Barr, A.M., Procyshyn, R.M., and Vila-Rodriguez, F. (2015). Response trajectories to clozapine in a secondary analysis of pivotal trials support using treatment response to subtype schizophrenia. *Can J Psychiatry* 60(3 Suppl 2)**,** S19-25.

Honer, W.G., Procyshyn, R.M., Chen, E.Y., MacEwan, G.W., and Barr, A.M. (2009). A translational research approach to poor treatment response in patients with schizophrenia: clozapine-antipsychotic polypharmacy. *J Psychiatry Neurosci* 34(6)**,** 433-442.

Honer, W.G., Thornton, A.E., Sherwood, M., MacEwan, G.W., Ehmann, T.S., Williams, R., et al. (2007). Conceptual and methodological issues in the design of clinical trials of antipsychotics for the treatment of schizophrenia. *CNS Drugs* 21(9)**,** 699-714.

Huang, W.L., Chang, L.R., Kuo, T.B., Lin, Y.H., Chen, Y.Z., and Yang, C.C. (2013). Impact of antipsychotics and anticholinergics on autonomic modulation in patients with schizophrenia. *J Clin Psychopharmacol* 33(2)**,** 170-177. doi: 10.1097/JCP.0b013e3182839052.

Huikuri, H.V., Niemela, M.J., Ojala, S., Rantala, A., Ikaheimo, M.J., and Airaksinen, K.E. (1994). Circadian rhythms of frequency domain measures of heart rate variability in healthy subjects and patients with coronary artery disease. Effects of arousal and upright posture. *Circulation* 90(1)**,** 121-126.

Ikezawa, S., Corbera, S., Liu, J., and Wexler, B.E. (2012). Empathy in electrodermal responsive and nonresponsive patients with schizophrenia. *Schizophrenia research* 142(1-3)**,** 71-76. doi: 10.1016/j.schres.2012.09.011.

Iqbal, M.M., Rahman, A., Husain, Z., Mahmud, S.Z., Ryan, W.G., and Feldman, J.M. (2003). Clozapine: a clinical review of adverse effects and management. *Annals of clinical psychiatry : official journal of the American Academy of Clinical Psychiatrists* 15(1)**,** 33-48.

Jänig, W. (2006). *The Integrative Action of the Autonomic Nervous System: Neurobiology of Homeostasis.* New York: Cambridge University Press.

John, A., Yeh, C., Boyd, J., and Greilich, P.E. (2010). Treatment of refractory hypotension with low-dose vasopressin in a patient receiving clozapine. *J Cardiothorac Vasc Anesth* 24(3)**,** 467-468. doi: S1053-0770(09)00338-3 [pii]

10.1053/j.jvca.2009.09.005.

Jones, W.R., Narayana, U., Howarth, S., Shinners, J., and Nazar, Q. (2014). Cardiovascular monitoring in patients prescribed clozapine. *Psychiatric bulletin* 38(3)**,** 140. doi: 10.1192/pb.38.3.140a.

Kalkman, H.O., Neumann, V., Hoyer, D., and Tricklebank, M.D. (1998). The role of alpha2-adrenoceptor antagonism in the anti-cataleptic properties of the atypical neuroleptic agent, clozapine, in the rat. *British journal of pharmacology* 124(7)**,** 1550-1556. doi: 10.1038/sj.bjp.0701975.

Kane, J., Honigfeld, G., Singer, J., and Meltzer, H. (1988). Clozapine for the treatment-resistant schizophrenic. A double-blind comparison with chlorpromazine. *Archives of general psychiatry* 45(9)**,** 789-796.

Kane, J.M., Cooper, T.B., Sachar, E.J., Halpern, F.S., and Bailine, S. (1981). Clozapine: plasma levels and prolactin response. *Psychopharmacology* 73(2)**,** 184-187.

Kar, N., Barreto, S., and Chandavarkar, R. (2016). Clozapine Monitoring in Clinical Practice: Beyond the Mandatory Requirement. *Clinical psychopharmacology and neuroscience : the official scientific journal of the Korean College of Neuropsychopharmacology* 14(4)**,** 323-329. doi: 10.9758/cpn.2016.14.4.323.

Kelly, D.L., McMahon, R.P., Liu, F., Love, R.C., Wehring, H.J., Shim, J.C., et al. (2010). Cardiovascular disease mortality in patients with chronic schizophrenia treated with clozapine: a retrospective cohort study. *The Journal of clinical psychiatry* 71(3)**,** 304-311. doi: 10.4088/JCP.08m04718yel.

Kilian, J.G., Kerr, K., Lawrence, C., and Celermajer, D.S. (1999). Myocarditis and cardiomyopathy associated with clozapine. *Lancet* 354(9193)**,** 1841-1845.

Kim, J.H., Yi, S.H., Yoo, C.S., Yang, S.A., Yoon, S.C., Lee, K.Y., et al. (2004). Heart rate dynamics and their relationship to psychotic symptom severity in clozapine-treated schizophrenic subjects. *Prog Neuropsychopharmacol Biol Psychiatry* 28(2)**,** 371-378. doi: S0278-5846(03)00312-9 [pii]

10.1016/j.pnpbp.2003.11.007.

Koren, W., Kreis, Y., Duchowiczny, K., Prince, T., Sancovici, S., Sidi, Y., et al. (1997). Lactic acidosis and fatal myocardial failure due to clozapine. *The Annals of pharmacotherapy* 31(2)**,** 168-170.

Krentz, A.J., Mikhail, S., Cantrell, P., and Hill, G.M. (2001). Drug Points: Pseudophaeochromocytoma syndrome associated with clozapine. *BMJ* 322(7296)**,** 1213.

Krisch, I., Bole-Vunduk, B., Pepelnak, M., Lavric, B., Ocvirk, A., Budihna, M.V., et al. (1994). Pharmacological studies with two new ergoline derivatives, the potential antipsychotics LEK-8829 and LEK-8841. *J Pharmacol Exp Ther* 271(1)**,** 343-352.

La Grenade, L., Graham, D., and Trontell, A. (2001). Myocarditis and cardiomyopathy associated with clozapine use in the United States. *The New England journal of medicine* 345(3)**,** 224-225. doi: 10.1056/NEJM200107193450317.

La Rovere, M.T., Bigger, J.T., Jr., Marcus, F.I., Mortara, A., and Schwartz, P.J. (1998). Baroreflex sensitivity and heart-rate variability in prediction of total cardiac mortality after myocardial infarction. ATRAMI (Autonomic Tone and Reflexes After Myocardial Infarction) Investigators. *Lancet* 351(9101)**,** 478-484. doi: S0140673697111448 [pii].

Lang, D.J., Barr, A.M., and Procyshyn, R.M. (2013). Management of Medication-Related Cardiometabolic Risk in Patients with Severe Mental Illness. *Curr Cardiovasc Risk Rep* 7(4)**,** 283-287.

Lee, L.H., White, R.F., Barr, A.M., Honer, W.G., and Procyshyn, R.M. (2016). Elevated clozapine plasma concentration secondary to a urinary tract infection: proposed mechanisms. *J Psychiatry Neurosci* 41(4)**,** E67-68.

Leo, R.J., Kreeger, J.L., and Kim, K.Y. (1996). Cardiomyopathy associated with clozapine. *The Annals of pharmacotherapy* 30(6)**,** 603-605.

Leung, J.G., Nelson, S., and Hocker, S. (2015). Failure of Induced Hypertension for Symptomatic Vasospasm in the Setting of Clozapine Therapy. *Neurocrit Care* 23(3)**,** 409-413. doi: 10.1007/s12028-015-0129-6

10.1007/s12028-015-0129-6 [pii].

Leung, J.Y., Barr, A.M., Procyshyn, R.M., Honer, W.G., and Pang, C.C. (2012). Cardiovascular side-effects of antipsychotic drugs: the role of the autonomic nervous system. *Pharmacology & therapeutics* 135(2)**,** 113-122. doi: 10.1016/j.pharmthera.2012.04.003.

Li, J.K., Yeung, V.T., Leung, C.M., Chow, C.C., Ko, G.T., So, W.Y., et al. (1997). Clozapine: a mimicry of phaeochromocytoma. *Aust N Z J Psychiatry* 31(6)**,** 889-891.

Lindstrom, L.H. (1988). The effect of long-term treatment with clozapine in schizophrenia: a retrospective study in 96 patients treated with clozapine for up to 13 years. *Acta Psychiatr Scand* 77(5)**,** 524-529.

Mackin, P. (2008). Cardiac side effects of psychiatric drugs. *Human psychopharmacology* 23 Suppl 1**,** 3-14. doi: 10.1002/hup.915.

Mansier, P., Clairambault, J., Charlotte, N., Medigue, C., Vermeiren, C., LePape, G., et al. (1996). Linear and non-linear analyses of heart rate variability: a minireview. *Cardiovascular research* 31(3)**,** 371-379.

Masri, B., Salahpour, A., Didriksen, M., Ghisi, V., Beaulieu, J.M., Gainetdinov, R.R., et al. (2008). Antagonism of dopamine D2 receptor/beta-arrestin 2 interaction is a common property of clinically effective antipsychotics. *Proceedings of the National Academy of Sciences of the United States of America* 105(36)**,** 13656-13661. doi: 10.1073/pnas.0803522105.

Masuo, K., Kawaguchi, H., Mikami, H., Ogihara, T., and Tuck, M.L. (2003). Serum uric acid and plasma norepinephrine concentrations predict subsequent weight gain and blood pressure elevation. *Hypertension* 42(4)**,** 474-480. doi: 10.1161/01.HYP.0000091371.53502.D3.

Mathewson, K.J., Jetha, M.K., Goldberg, J.O., and Schmidt, L.A. (2012). Autonomic regulation predicts performance on Wisconsin Card Sorting Test (WCST) in adults with schizophrenia. *Biol Psychol* 91(3)**,** 389-399. doi: S0301-0511(12)00190-1 [pii]

10.1016/j.biopsycho.2012.09.002.

Mayer, A.F., Schroeder, C., Heusser, K., Tank, J., Diedrich, A., Schmieder, R.E., et al. (2006). Influences of norepinephrine transporter function on the distribution of sympathetic activity in humans. *Hypertension* 48(1)**,** 120-126. doi: 10.1161/01.HYP.0000225424.13138.5d.

McEvoy, J.P., Meyer, J.M., Goff, D.C., Nasrallah, H.A., Davis, S.M., Sullivan, L., et al. (2005). Prevalence of the metabolic syndrome in patients with schizophrenia: baseline results from the Clinical Antipsychotic Trials of Intervention Effectiveness (CATIE) schizophrenia trial and comparison with national estimates from NHANES III. *Schizophrenia research* 80(1)**,** 19-32. doi: 10.1016/j.schres.2005.07.014.

Melkersson, K. (2005). Differences in prolactin elevation and related symptoms of atypical antipsychotics in schizophrenic patients. *The Journal of clinical psychiatry* 66(6)**,** 761-767.

Meltzer, H.Y. (1997). Treatment-resistant schizophrenia--the role of clozapine. *Current medical research and opinion* 14(1)**,** 1-20. doi: 10.1185/03007999709113338.

Meltzer, H.Y. (2012). Clozapine: balancing safety with superior antipsychotic efficacy. *Clinical schizophrenia & related psychoses* 6(3)**,** 134-144. doi: 10.3371/CSRP.6.3.5.

Meltzer, H.Y., and Okayli, G. (1995). Reduction of suicidality during clozapine treatment of neuroleptic-resistant schizophrenia: impact on risk-benefit assessment. *The American journal of psychiatry* 152(2)**,** 183-190. doi: 10.1176/ajp.152.2.183.

Merrill, D.B., Ahmari, S.E., Bradford, J.M., and Lieberman, J.A. (2006). Myocarditis during clozapine treatment. *The American journal of psychiatry* 163(2)**,** 204-208. doi: 10.1176/appi.ajp.163.2.204.

Merrill, D.B., Dec, G.W., and Goff, D.C. (2005). Adverse cardiac effects associated with clozapine. *Journal of clinical psychopharmacology* 25(1)**,** 32-41.

Modai, I., Hirschmann, S., Rava, A., Kurs, R., Barak, P., Lichtenberg, P., et al. (2000). Sudden death in patients receiving clozapine treatment: a preliminary investigation. *Journal of clinical psychopharmacology* 20(3)**,** 325-327.

Monsma, F.J., Jr., Shen, Y., Ward, R.P., Hamblin, M.W., and Sibley, D.R. (1993). Cloning and expression of a novel serotonin receptor with high affinity for tricyclic psychotropic drugs. *Molecular pharmacology* 43(3)**,** 320-327.

Mueck-Weymann, M., Rechlin, T., Ehrengut, F., Rauh, R., Acker, J., Dittmann, R.W., et al. (2002). Effects of olanzapine and clozapine upon pulse rate variability. *Depress Anxiety* 16(3)**,** 93-99. doi: 10.1002/da.10037.

Nasrallah, H.A. (2008). Atypical antipsychotic-induced metabolic side effects: insights from receptor-binding profiles. *Molecular psychiatry* 13(1)**,** 27-35. doi: 10.1038/sj.mp.4002066.

Nielsen, B.M., Mehlsen, J., and Behnke, K. (1988). Altered balance in the autonomic nervous system in schizophrenic patients. *Clin Physiol* 8(2)**,** 193-199.

Nilsson, B.M., Holm, G., Hultman, C.M., and Ekselius, L. (2015). Cognition and autonomic function in schizophrenia: inferior cognitive test performance in electrodermal and niacin skin flush non-responders. *European psychiatry : the journal of the Association of European Psychiatrists* 30(1)**,** 8-13. doi: 10.1016/j.eurpsy.2014.06.004.

Nolan, J., Batin, P.D., Andrews, R., Lindsay, S.J., Brooksby, P., Mullen, M., et al. (1998). Prospective study of heart rate variability and mortality in chronic heart failure: results of the United Kingdom heart failure evaluation and assessment of risk trial (UK-heart). *Circulation* 98(15)**,** 1510-1516.

Ohman, A. (1981). Electrodermal activity and vulnerability to schizophrenia: a review. *Biological psychology* 12(2-3)**,** 87-145.

Olfson, M., Marcus, S.C., Corey-Lisle, P., Tuomari, A.V., Hines, P., and L'Italien, G.J. (2006). Hyperlipidemia following treatment with antipsychotic medications. *The American journal of psychiatry* 163(10)**,** 1821-1825. doi: 10.1176/ajp.2006.163.10.1821.

Oyewumi, L.K., Cernovsky, Z.Z., and Freeman, D.J. (2004). Autonomic signs and dosing during the initial stages of clozapine therapy. *Med Sci Monit* 10(2)**,** PI19-23.

Paton, J.F., Boscan, P., Pickering, A.E., and Nalivaiko, E. (2005). The yin and yang of cardiac autonomic control: vago-sympathetic interactions revisited. *Brain Res Brain Res Rev* 49(3)**,** 555-565. doi: 10.1016/j.brainresrev.2005.02.005.

Pereira, Y.D., Srivastava, A., Cuncoliencar, B.S., and Naik, N. (2010). Resolution of symptoms in neuroleptic malignant syndrome. *Indian journal of psychiatry* 52(3)**,** 264-266. doi: 10.4103/0019-5545.70988.

Pickar, D., Owen, R.R., Litman, R.E., Konicki, E., Gutierrez, R., and Rapaport, M.H. (1992). Clinical and biologic response to clozapine in patients with schizophrenia. Crossover comparison with fluphenazine. *Archives of general psychiatry* 49(5)**,** 345-353.

Prasad, S.E., and Kennedy, H.G. (2003). Pseudophaeochromocytoma associated with clozapine treatment. *Irish Journal of psychological medicine* 20(4)**,** 132-134.

Procyshyn, R.M., Honer, W.G., and Barr, A.M. (2009). Do serum lipids predict response to clozapine treatment? *J Psychiatry Neurosci* 34(2)**,** 168.

Procyshyn, R.M., Honer, W.G., Wu, T.K., Ko, R.W., McIsaac, S.A., Young, A.H., et al. (2010). Persistent antipsychotic polypharmacy and excessive dosing in the community psychiatric treatment setting: a review of medication profiles in 435 Canadian outpatients. *J Clin Psychiatry* 71(5)**,** 566-573.

Procyshyn, R.M., Vila-Rodriguez, F., Honer, W.G., and Barr, A.M. (2014). Clozapine administered once versus twice daily: does it make a difference? *Med Hypotheses* 82(2)**,** 225-228. doi: 10.1016/j.mehy.2013.11.043.

Procyshyn, R.M., Wasan, K.M., Thornton, A.E., Barr, A.M., Chen, E.Y., Pomarol-Clotet, E., et al. (2007). Changes in serum lipids, independent of weight, are associated with changes in symptoms during long-term clozapine treatment. *J Psychiatry Neurosci* 32(5)**,** 331-338.

Quintana, D.S., Westlye, L.T., Kaufmann, T., Rustan, O.G., Brandt, C.L., Haatveit, B., et al. (2016). Reduced heart rate variability in schizophrenia and bipolar disorder compared to healthy controls. *Acta Psychiatr Scand* 133(1)**,** 44-52. doi: 10.1111/acps.12498.

Ray, W.A., Chung, C.P., Murray, K.T., Hall, K., and Stein, C.M. (2009). Atypical antipsychotic drugs and the risk of sudden cardiac death. *The New England journal of medicine* 360(3)**,** 225-235. doi: 10.1056/NEJMoa0806994.

Rechlin, T., Beck, G., Weis, M., and Kaschka, W.P. (1998). Correlation between plasma clozapine concentration and heart rate variability in schizophrenic patients. *Psychopharmacology* 135**,** 338-441.

Rechlin, T., Claus, D., and Weis, M. (1994). Heart rate variability in schizophrenic patients and changes of autonomic heart rate parameters during treatment with clozapine. *Biol Psychiatry* 35(11)**,** 888-892.

Reinstein, M.J., Chasonov, M.A., Colombo, K.D., Jones, L.E., and Sonnenberg, J.G. (2002). Reduction of Suicidality in Patients with Schizophrenia Receiving Clozapine. *Clinical Drug Investigation* 22(5)**,** 341-346. doi: 10.2165/00044011-200222050-00008.

Richelson, E., and Souder, T. (2000). Binding of antipsychotic drugs to human brain receptors focus on newer generation compounds. *Life sciences* 68(1)**,** 29-39.

Richman, J.S., and Moorman, J.R. (2000). Physiological time-series analysis using approximate entropy and sample entropy. *American journal of physiology. Heart and circulatory physiology* 278(6)**,** H2039-2049. doi: 10.1152/ajpheart.2000.278.6.H2039.

Romo-Nava, F., Alvarez-Icaza Gonzalez, D., Fresan-Orellana, A., Saracco Alvarez, R., Becerra-Palars, C., Moreno, J., et al. (2014). Melatonin attenuates antipsychotic metabolic effects: an eight-week randomized, double-blind, parallel-group, placebo-controlled clinical trial. *Bipolar disorders* 16(4)**,** 410-421. doi: 10.1111/bdi.12196.

Ronaldson, K.J., Fitzgerald, P.B., and McNeil, J.J. (2015). Clozapine-induced myocarditis, a widely overlooked adverse reaction. *Acta psychiatrica Scandinavica* 132(4)**,** 231-240. doi: 10.1111/acps.12416.

Ronaldson, K.J., Fitzgerald, P.B., Taylor, A.J., Topliss, D.J., and McNeil, J.J. (2011). A new monitoring protocol for clozapine-induced myocarditis based on an analysis of 75 cases and 94 controls. *The Australian and New Zealand journal of psychiatry* 45(6)**,** 458-465. doi: 10.3109/00048674.2011.572852.

Ronaldson, K.J., Taylor, A.J., Fitzgerald, P.B., Topliss, D.J., Elsik, M., and McNeil, J.J. (2010). Diagnostic characteristics of clozapine-induced myocarditis identified by an analysis of 38 cases and 47 controls. *J Clin Psychiatry* 71(8)**,** 976-981. doi: 10.4088/JCP.09m05024yel.

Rumantir, M.S., Kaye, D.M., Jennings, G.L., Vaz, M., Hastings, J.A., and Esler, M.D. (2000). Phenotypic evidence of faulty neuronal norepinephrine reuptake in essential hypertension. *Hypertension* 36(5)**,** 824-829.

Safferman, A., Lieberman, J.A., Kane, J.M., Szymanski, S., and Kinon, B. (1991). Update on the clinical efficacy and side effects of clozapine. *Schizophrenia bulletin* 17(2)**,** 247-261.

Sara, J., Jenkins, M., Chohan, T., Jolly, K., Shepherd, L., Gandhi, N.Y., et al. (2013). Clozapine use presenting with pseudopheochromocytoma in a schizophrenic patient: a case report. *Case Rep Endocrinol* 2013**,** 194927. doi: 10.1155/2013/194927.

Schell, A.M., Dawson, M.E., Rissling, A., Ventura, J., Subotnik, K.L., Gitlin, M.J., et al. (2005). Electrodermal predictors of functional outcome and negative symptoms in schizophrenia. *Psychophysiology* 42(4)**,** 483-492. doi: 10.1111/j.1469-8986.2005.00300.x.

Schlaich, M.P., Kaye, D.M., Lambert, E., Sommerville, M., Socratous, F., and Esler, M.D. (2003). Relation between cardiac sympathetic activity and hypertensive left ventricular hypertrophy. *Circulation* 108(5)**,** 560-565. doi: 10.1161/01.CIR.0000081775.72651.B6.

Schlaich, M.P., Lambert, E., Kaye, D.M., Krozowski, Z., Campbell, D.J., Lambert, G., et al. (2004). Sympathetic augmentation in hypertension: role of nerve firing, norepinephrine reuptake, and Angiotensin neuromodulation. *Hypertension* 43(2)**,** 169-175. doi: 10.1161/01.HYP.0000103160.35395.9E.

Seddigh, R., Keshavarz-Akhlaghi, A.A., and Shariati, B. (2014). Treating methamphetamine-induced resistant psychosis with clozapine. *Case Rep Psychiatry* 2014**,** 845145. doi: 10.1155/2014/845145.

Shannon, J.R., Flattem, N.L., Jordan, J., Jacob, G., Black, B.K., Biaggioni, I., et al. (2000). Orthostatic intolerance and tachycardia associated with norepinephrine-transporter deficiency. *The New England journal of medicine* 342(8)**,** 541-549. doi: 10.1056/NEJM200002243420803.

Sheldon, R.S., Grubb, B.P., 2nd, Olshansky, B., Shen, W.K., Calkins, H., Brignole, M., et al. (2015). 2015 heart rhythm society expert consensus statement on the diagnosis and treatment of postural tachycardia syndrome, inappropriate sinus tachycardia, and vasovagal syncope. *Heart Rhythm* 12(6)**,** e41-63. doi: 10.1016/j.hrthm.2015.03.029.

Shen, Y., Monsma, F.J., Jr., Metcalf, M.A., Jose, P.A., Hamblin, M.W., and Sibley, D.R. (1993). Molecular cloning and expression of a 5-hydroxytryptamine7 serotonin receptor subtype. *The Journal of biological chemistry* 268(24)**,** 18200-18204.

Spivak, B., Roitman, S., Vered, Y., Mester, R., Graff, E., Talmon, Y., et al. (1998). Diminished suicidal and aggressive behavior, high plasma norepinephrine levels, and serum triglyceride levels in chronic neuroleptic-resistant schizophrenic patients maintained on clozapine. *Clinical neuropharmacology* 21(4)**,** 245-250.

Stein, P.K., Bosner, M.S., Kleiger, R.E., and Conger, B.M. (1994). Heart rate variability: a measure of cardiac autonomic tone. *American heart journal* 127(5)**,** 1376-1381.

Sztajzel, J. (2004). Heart rate variability: a noninvasive electrocardiographic method to measure the autonomic nervous system. *Swiss medical weekly* 134(35-36)**,** 514-522. doi: 2004/35/smw-10321.

Tang, V.M., Lang, D.J., Giesbrecht, C.J., Panenka, W.J., Willi, T., Procyshyn, R.M., et al. (2015). White matter deficits assessed by diffusion tensor imaging and cognitive dysfunction in psychostimulant users with comorbid human immunodeficiency virus infection. *BMC Res Notes* 8**,** 515. doi: 10.1186/s13104-015-1501-5.

Thomas, J.A., and Marks, B.H. (1978). Plasma norepinephrine in congestive heart failure. *The American journal of cardiology* 41(2)**,** 233-243.

Thomas, L., and Pollak, P.T. (2003). Delayed recovery associated with persistent serum concentrations after clozapine overdose. *The Journal of emergency medicine* 25(1)**,** 61-66.

Thornton, A.E., Procyshyn, R.M., Barr, A.M., MacEwan, G.W., and Honer, W.G. (2015). Cognition and Plasma Ratio of Clozapine to N-desmethylclozapine in Patients With Clozapine-Resistant Schizophrenia. *Am J Psychiatry* 172(12)**,** 1259. doi: 10.1176/appi.ajp.2015.15070899.

Triposkiadis, F., Karayannis, G., Giamouzis, G., Skoularigis, J., Louridas, G., and Butler, J. (2009). The sympathetic nervous system in heart failure physiology, pathophysiology, and clinical implications. *J Am Coll Cardiol* 54(19)**,** 1747-1762. doi: S0735-1097(09)01689-1 [pii]

10.1016/j.jacc.2009.05.015.

Tse, L., Barr, A.M., Scarapicchia, V., and Vila-Rodriguez, F. (2015). Neuroleptic Malignant Syndrome: A Review from a Clinically Oriented Perspective. *Curr Neuropharmacol* 13(3)**,** 395-406.

Tsuji, H., Larson, M.G., Venditti, F.J., Jr., Manders, E.S., Evans, J.C., Feldman, C.L., et al. (1996). Impact of reduced heart rate variability on risk for cardiac events. The Framingham Heart Study. *Circulation* 94(11)**,** 2850-2855.

Tümüklü, M.N., Alptekin, K., Kırımlı, Ö., Aslan, Ö., Akdede, B.B., Badak, Ö., et al. (2008). Arrhythmic Markers and Clozapine in Patients with Schizophrenia: Effect of 10 weeks Clozapine Treatment on Heart Rate Variability, Late Potentials and QT Dispersion. *Klinik Psikofarmakoloji Bülteni* 18**,** 167-173.

Van Tol, H.H., Bunzow, J.R., Guan, H.C., Sunahara, R.K., Seeman, P., Niznik, H.B., et al. (1991). Cloning of the gene for a human dopamine D4 receptor with high affinity for the antipsychotic clozapine. *Nature* 350(6319)**,** 610-614. doi: 10.1038/350610a0.

Vila-Rodriguez, F., Tsang, P., and Barr, A.M. (2013). Chronic benign neutropenia/agranulocytosis associated with non-clozapine antipsychotics. *Am J Psychiatry* 170(10)**,** 1213-1214. doi: 10.1176/appi.ajp.2013.13020215.

Walker, A.M., Lanza, L.L., Arellano, F., and Rothman, K.J. (1997). Mortality in current and former users of clozapine. *Epidemiology* 8(6)**,** 671-677.

Wang, J.F., Min, J.Y., Hampton, T.G., Amende, I., Yan, X., Malek, S., et al. (2008). Clozapine-induced myocarditis: role of catecholamines in a murine model. *Eur J Pharmacol* 592(1-3)**,** 123-127. doi: S0014-2999(08)00666-3 [pii]

10.1016/j.ejphar.2008.06.088.

Wang, Y.C., Chen, C.Y., Kuo, T.B., Lai, C.J., and Yang, C.C. (2012). Influence of antipsychotic agents on heart rate variability in male WKY rats: implications for cardiovascular safety. *Neuropsychobiology* 65(4)**,** 216-226. doi: 000337459 [pii]

10.1159/000337459.

Wheeler, T., and Watkins, P.J. (1973). Cardiac denervation in diabetes. *Br Med J* 4(5892)**,** 584-586.

Whitney, Z., Procyshyn, R.M., Fredrikson, D.H., and Barr, A.M. (2015). Treatment of clozapine-associated weight gain: a systematic review. *Eur J Clin Pharmacol* 71(4)**,** 389-401. doi: 10.1007/s00228-015-1807-1.

Willi, T.S., Barr, A.M., Gicas, K., Lang, D.J., Vila-Rodriguez, F., Su, W., et al. (2016a). Characterization of white matter integrity deficits in cocaine-dependent individuals with substance-induced psychosis compared with non-psychotic cocaine users. *Addict Biol*. doi: 10.1111/adb.12363.

Willi, T.S., Lang, D.J., Honer, W.G., Smith, G.N., Thornton, A.E., Panenka, W.J., et al. (2016b). Subcortical grey matter alterations in cocaine dependent individuals with substance-induced psychosis compared to non-psychotic cocaine users. *Schizophr Res* 176(2-3)**,** 158-163. doi: 10.1016/j.schres.2016.08.001.

Xhyheri, B., Manfrini, O., Mazzolini, M., Pizzi, C., and Bugiardini, R. (2012). Heart rate variability today. *Progress in cardiovascular diseases* 55(3)**,** 321-331. doi: 10.1016/j.pcad.2012.09.001.

Yacoub, A., and Francis, A. (2006). Neuroleptic malignant syndrome induced by atypical neuroleptics and responsive to lorazepam. *Neuropsychiatric disease and treatment* 2(2)**,** 235-240.

Young, C.R., Bowers, M.B., Jr., and Mazure, C.M. (1998). Management of the adverse effects of clozapine. *Schizophrenia bulletin* 24(3)**,** 381-390.

Zahn, T.P., and Pickar, D. (1993). Autonomic effects of clozapine in schizophrenia: comparison with placebo and fluphenazine. *Biol Psychiatry* 34(1-2)**,** 3-12. doi: 0006-3223(93)90250-H [pii].

Zoccali, C., Mallamaci, F., Tripepi, G., Parlongo, S., Cutrupi, S., Benedetto, F.A., et al. (2002). Norepinephrine and concentric hypertrophy in patients with end-stage renal disease. *Hypertension* 40(1)**,** 41-46.
